# Targeting WTAP/ROR1/WNT5A‐Mediated Crosstalk Between Glioma Stem Cells and Macrophages to Normalize Tumor Vasculature and Enhance Chemotherapy

**DOI:** 10.1002/advs.202520661

**Published:** 2026-01-21

**Authors:** Xiaoyong Chen, Bo Pang, Yun Liu, Jiangwei Wang, Lanhui Zheng, Yuzhou Chang, Yingxuan Sun, Haoyuan Sun, Huiyuan Chen, Jiawei Cai, Zanyi Wu, Qing Chang, Yongzhi Wang, Dezhi Kang, Tao Jiang, Ruichao Chai

**Affiliations:** ^1^ Department of Neurosurgery Beijing Tiantan Hospital Capital Medical University Beijing China; ^2^ Department of Neurosurgery Neurosurgery Research Institute The First Affiliated Hospital Fujian Medical University Fuzhou China; ^3^ Beijing Neurosurgical Institute Capital Medical University Beijing China; ^4^ Department of Neurosurgery National Regional Medical Center Binhai Campus of the First Affiliated Hospital Fujian Medical University Fuzhou China; ^5^ College of Basic Medical Sciences Dalian Medical University Dalian China; ^6^ Beijing Key Laboratory of Drug Innovation For Neuro‐Oncology Beijing China; ^7^ Beijing Engineering Research Center of Targeted Drugs and Cell Therapy For CNS Tumors Beijing China; ^8^ Chinese Glioma Genome Atlas Network (CGGA) and Asian Glioma Genome Atlas Network (AGGA) Beijing China

**Keywords:** endothelial differentiation, glioma stem cell, m^6^A, macrophage, targeted therapy

## Abstract

Glioma stem cells (GSCs) can trans‐differentiate into glioma‐derived endothelial cells (GDECs), contributing to glioblastoma progression and therapy resistance. The molecular basis underpinning GSC‐GDEC differentiation remain incompletely elucidated. Here, ROR1 is identified as a key regulator of GSC‐to‐GDEC differentiation under hypoxia. ROR1 expression is elevated in GDECs across patient‐derived cells, xenografts, and tumor samples, confirmed by single‐cell multiomics, Western blotting, and multiplex staining. Mechanistically, WNT5A, secreted by tumor‐associated macrophages (TAMs) under hypoxia, activates ROR1‐mediated Wingless‐type MMTV integration site family (WNT) signaling in GSCs, promoting GDEC formation. Interestingly, hypoxia‐induced WTAP not only enhances ROR1 stability through m^6^A modification in a HuR‐dependent manner, but also contributes to WNT5A+TAMs infiltration. Clinically, high ROR1 and WNT5A expression correlates with poor glioblastoma prognosis, and WNT5A may serve as a circulating biomarker. Therapeutically, targeting ROR1 via endothelial‐specific Adno‐Associated Virus (AAV) knockdown or the antibody‐drug conjugate Zilovertamab vedotin (VLS‐101) normalizes vasculature, improves temozolomide delivery, and sensitizes tumors in glioblastoma organoids and xenografts. These findings highlight the ROR1–WNT5A axis as a promising target in glioblastomas treatment.

## Graphical abstract

In hypoxic microenvironment, WNT5A is predominantly secreted by tumor‐associated macrophages. Hypoxia‐induced WTAP mediates ROR1 stability by m^6^A modifications in a HuR‐dependent manner in Glioma stem cells (GSCs). WNT5A activates the WNT pathway via ROR1 binding on GSCs, driving glioma‐derived endothelial cells (GDECs) differentiation. Targeting ROR1 with VLS‐101 inhibits GDEC formation, promotes vascular normalization, and enhances temozolomide efficacy.

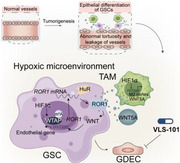

## 1 Introduction

Glioblastoma (GBM) is the most prevalent primary intracranial tumors in adults, along with necrosis, microvascular proliferation, and a hypoxia microenvironment [1, 2, 3, 4]. Despite the current standard therapeutic regimen comprising surgical resection followed by adjuvant radiotherapy and chemotherapy, GBM patients continue to exhibit dismal clinical outcomes, with a median overall survival of merely 15–18 months [5, 6]. Therapeutic resistance and recurrence in GBM are closely linked to its hallmark pathological features, such as high invasiveness, profound intratumoral heterogeneity, a complex tumor microenvironment (TME), and the presence of the blood–brain barrier (BBB) and blood–tumor barrier (BTB) which restrict drug delivery [7].

GBM is characterized by high vascularization, and the aberrant vascularization in tumors typically induces intratumorally hypoxia, acidic pH conditions, and compromised drug delivery to TME [8, 9, 10]. Therefore, antiangiogenic therapy can disrupt GBM progression by suppressing neovascularization and impairing oxygen and nutrient supply [11]. However, clinically satisfactory outcomes have not yet been achieved with antiangiogenic targeted therapy. Glioma stem cells (GSCs) can differentiate into glioma‐derived endothelial cells (GDECs) and participate in vasculogenic mimicry through migration and morphological transformation, thereby establishing tumor‐nourishing vascular networks [8, 12, 13, 14]. Unlike normal vessels, these aberrant vascular in GBM showed structurally destabilized characteristics, and thus resulted in severely retard blood perfusion and very low drug permeability [9, 15]. Tumor vessel normalization has been an emerging strategy to enhance drug delivery efficacy in glioma [8]. Therefore, targeting molecules that selectively regulate aberrant glioma vasculature via GDEC without affecting the healthy blood–brain barrier could enhance intratumoral drug penetration and synergize with antitumor therapies.

Hypoxia serves as a hallmark feature of gliomas [10]. Emerging evidence including our work and others indicates that the hypoxic microenvironment induces tumor cell state transition and differentiation, while driving dynamic remodeling of the immune landscape [16, 17, 18]. Tumor‐associated macrophages (TAMs) represent the predominant immune cell population within the glioma microenvironment [19, 20, 21]. TAMs also directly interact with tumor cells to promote oncogenesis and angiogenesis [22]. A recent study reported that glioma‐associated macrophages secrete adrenomedullin to induce aberrant tumor angiogenesis [9]. In addition, hypoxia‐upregulated myeloid‐derived suppressor cells spatially colocalize with metabolically stem‐like tumor cells within pseudopalisading niches and interact with glioma stem cells, thereby driving tumor progression [23]. However, whether TAMs participate in GSC‐to‐GDEC differentiation and the underlying mechanisms remain elusive.

In this study, we systematically characterized the transcriptional reprogramming during GSC‐to‐GDEC differentiation, identifying ROR1 as the key molecular involved in this process. Single‐cell multiomics analysis revealed WNT5A as a hypoxia‐induced secretory factor predominantly produced by TAMs in gliomas. Functional studies established WNT5A as a ROR1 ligand that activates Wingless‐type MMTV integration site family (WNT) signaling to promote endothelial‐like differentiation of GSCs. Hypoxic microenvironment rewires the epitranscriptomic landscape in GSCs, where WTAP‐mediated m^6^A modifications cooperate with HuR to stabilize ROR1 mRNA, licensing stem cell‐derived vasculogenesis. In orthotopic glioma models, we demonstrated that both endothelial‐specific Adno‐Associated Virus (AAV)‐mediated ROR1 knockdown and antibody drug conjugate Zilovertamab vedotin (VLS‐101) targeting ROR1 improved vascular integrity of tumor, enhanced temozolomide (TMZ) accumulation specifically in tumor tissue (but not in normal brain), and thus potentiated temozolomide's tumoricidal efficacy, and prolonged survival in glioma‐bearing mice. Collectively, our study identifies ROR1 as a potential therapeutic target for inducing vascular normalization in glioma, while demonstrating the clinical translational potential of VLS‐101 for improving drug delivery and chemo‐sensitization in GBM.

## 2 Results

### 2.1 ROR1 is Upregulated in GDECs Within Hypoxic Regions of GBM

**FIGURE 1:**
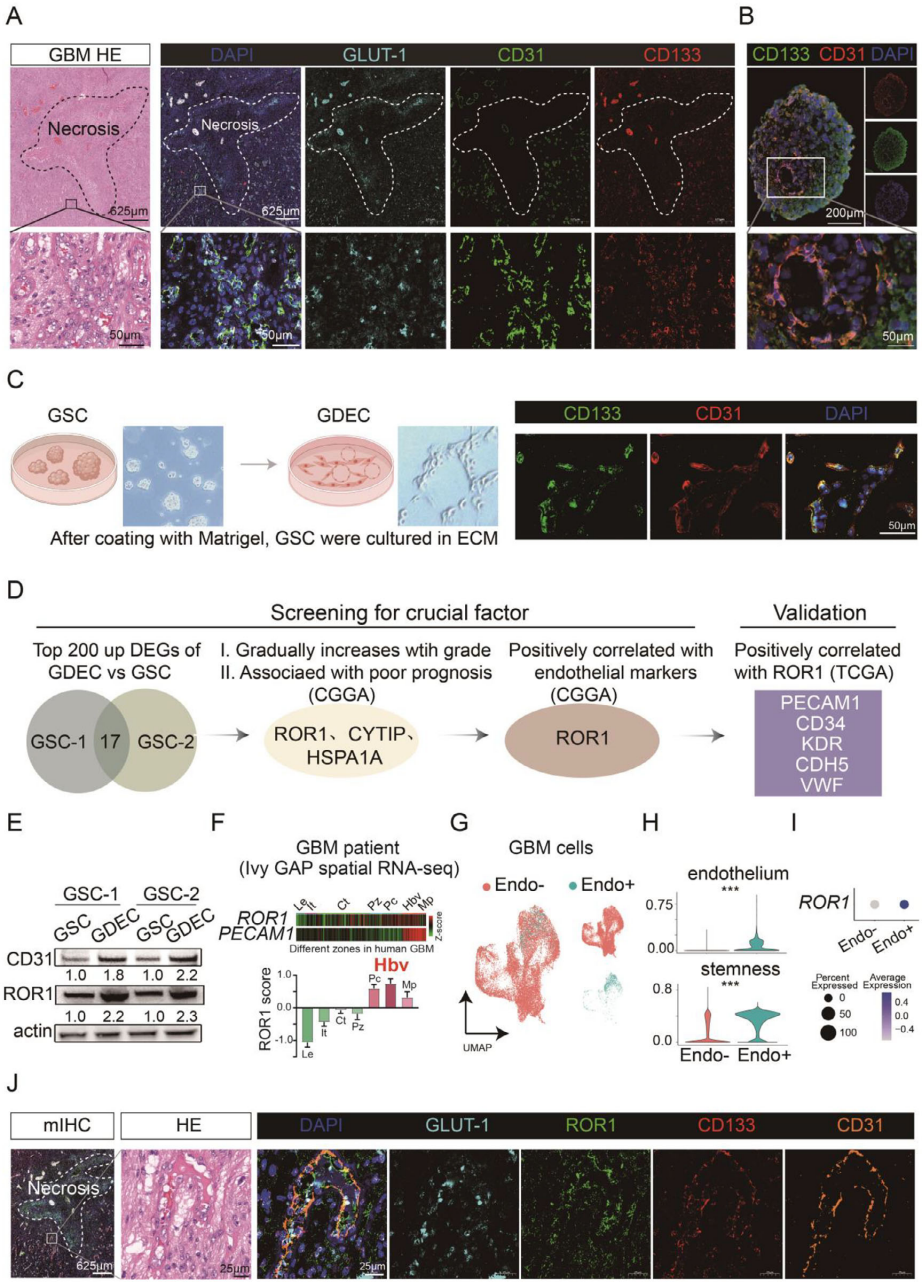
Identification of GDEC related genes in GBM patient samples and patient‐derived GSCs. (A) H&E and multiplex immunofluorescence staining of tissue section from GBM patient. The hypoxic necrotic region is outlined by black dotted lines (left). Multiplex immunofluorescence of adjacent GBM tissue section and the hypoxic necrotic zone is demarcated by white dotted lines, with colocalization of CD31 and CD133 observed in the peri‐necrotic region (right). (B) Immunofluorescence staining of CD31, CD133, and DAPI in GBM patient‐derived organoids. (C) Schematic of in vitro GSC differentiation into GDEC (left). Tube formation assay and CD133/CD31 immunofluorescence co‐staining of GDEC (right). (D) Workflow for identifying GDEC drivers. Two GSC lines were differentiated into GDEC for RNA‐seq. The intersection of top 200 upregulated genes was analyzed for correlations with glioma grade, prognosis, and endothelial gene expression, yielding candidate gene ROR1, which was validated in TCGA database for endothelial gene associations. (E) Western blot analysis of CD31 and ROR1 protein expression changes in GDECs derived from two GSC lines. (F) Heatmap (top) and bar chart (bottom) of expression level of ROR1 and PECAM1 in Ivy GAP spatial RNA‐seq from GBM patients. (G) Tumor cells in scRNA‐seq from 6 GBM patients were classified into Endo− and Endo+ subsets based on endothelial gene expression. (H) Violin plots showing expression of endothelial genes, and stemness genes in Endo− and Endo+ cells. (I) Dot plot showing ROR1 expression in Endo− and Endo+ cells. (J) Multiplex immunohistochemistry of adjacent GBM tissue section (left) with the hypoxic necrotic zone demarcated by white dotted lines. Representative higher‐magnification HE‐stained image (middle) and higher‐magnification multiplex immunohistochemistry image (right) with colocalization of CD31, CD133, and ROR1 observed in the peri‐necrotic region.

In peri‐necrotic regions of human GBM tissues, we observed that a significant number of CD133+/CD31+ endothelial cells were localized around the GLUT‐1 signal, suggesting the active transition of GSCs into GDECs of GBM in hypoxic areas (Figure 1A). The CD133+/CD31+ cells were also detected in GBM‐derived organoids (Figure 1B). Next, to explore key molecular mechanisms underlying the transition of GSCs to GDECs, we constructed GDECs models using two paitent‐derived cells (PDCs) of GBM (Figure 1C). Then, we compared transcriptomic differences between GDECs and their corresponding GSCs using RNA sequencing (Figure S1A). We identified 17 genes that were upregulated in GDECs derived from both PDCs by intersecting their top 200 upregulated genes (Data S1,S2 and Figure 1D). Among these 17 genes, ROR1, CYTIP, and HSPA1A were positively correlated with histological grade and poorer prognosis of diffuse gliomas in two independent datasets from CGGA [24] (Figure 1D and Figure S1B–S1E). Notably, among these three genes, only ROR1 exhibited a positive correlation with the expression of endothelial cell markers in gliomas (Figure S1F). The positive correlation between expression of ROR1 and endothelial cell marker was also observed in the Cancer Genome Atlas (TCGA) glioma dataset (Figure S1G).

We then confirmed that both ROR1 mRNA and protein expression were upregulated in GDECs induced from GSCs (Figure 1E and Figure S2A). In our newly collected 9 glioma tissues, we observed that both ROR1 mRNA and protein expression levels increased with increasing tumor histological grades (Figure S2B and S2C). In public ATAC‐sequencing data, we detected chromatin accessibility signals for ROR1 in GSCs and GBM tissues, but not in tissues from lower‐grade gliomas (Figure S2D). Spatially, using RNA‐sequencing data of different tumor regions from the Ivy Glioblastoma Atlas Project [25], we found that ROR1 exhibited higher expression in the perinecrotic core (Pc), hypervascular border (Hbv), and marginal proliferative (Mp) zones, with particularly pronounced expression in Hbv regions (Figure 1F and Figure S2E).

At the single‐cell level, we reclustered malignant cells into Endo+ and Endo‐ populations based on the expression of endothelial markers PECAM1 and CD34 (Figure 1G and Figure S2F,S2G) [26, 27], using our previously published scRNA‐seq data of GBM tissues from six patients [20]. Interestingly, consist with endothelial genes trend (*p* < 0.001, Figure 1H and Figure S2H), Endo+ GBM cells exhibited significantly higher expression of stemness genes (*p* < 0.001) and ROR1 compared to Endo‐ GBM cells (Figure 1H,I and Figure S2I). To assess the expression of ROR1 in angiogenic regions of GBM tissue, we performed multiplex staining for ROR1, CD31, CD133, and GLUT‐1 on sections adjacent to those shown in Figure 1A. The results revealed that ROR1 was highly expressed in CD133+/CD31+ GDEC cells, which were localized in hypoxic regions (marked by higher GLUT‐1 expression) surrounding necrotic areas (indicated by white dotted lines) in GBM tissues from patients (Figure 1J and Figure S2J,S2K).

Collectively, these findings indicate that ROR1 is upregulated in GDECs within hypoxic regions and may play a critical role in promoting angiogenesis in these areas.

### 2.2 ROR1 Promotes the Formation of GDECs and Drives Malignant Progression in GBM

**FIGURE 2:**
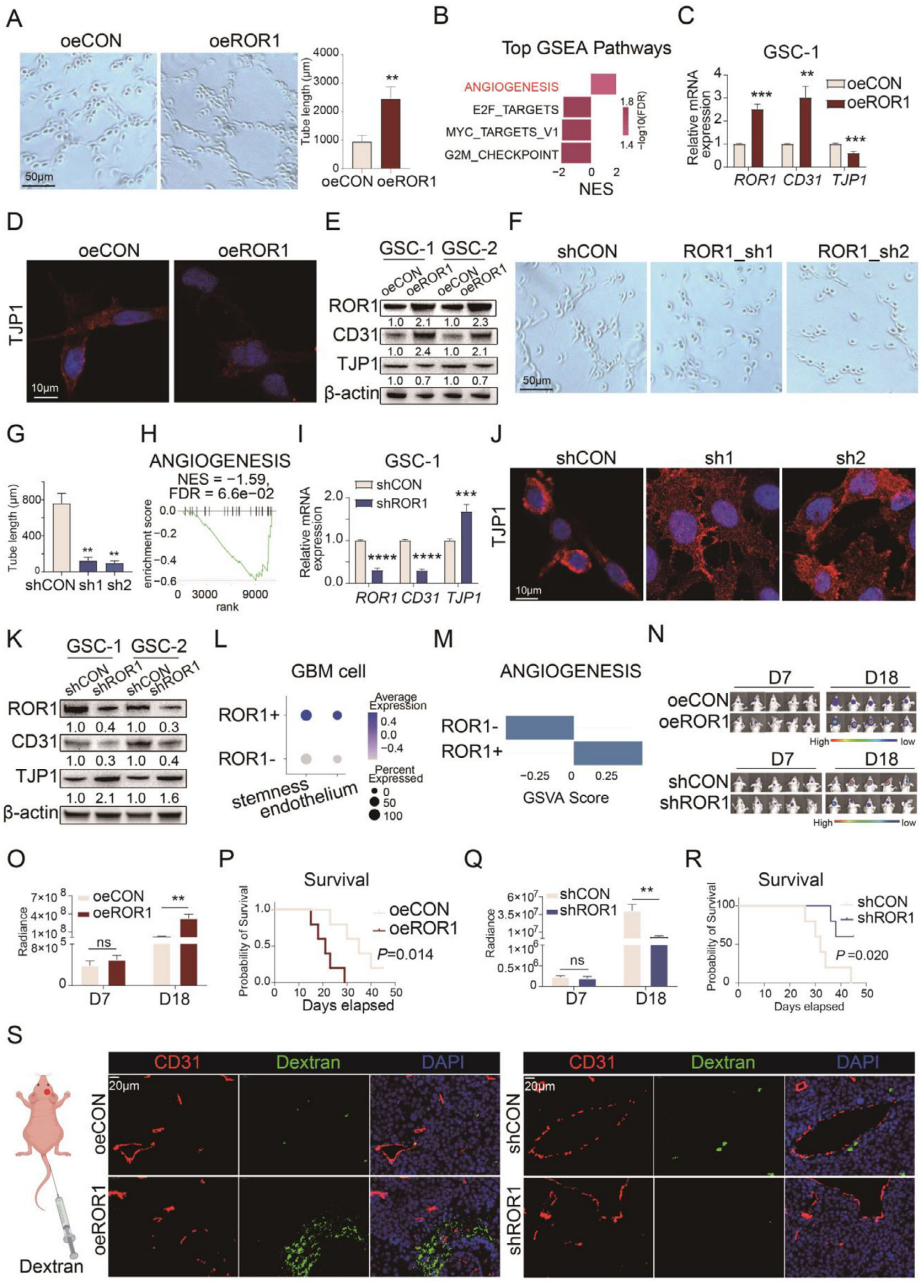
ROR1 modulates GDEC differentiation and malignant progression. (A) The images (left) and quantitative comparison of tube length (right) between oeROR1 and oeCON GSCs in tube formation assay (*n* = 3). (B) Top enriched GSEA pathways in differential gene analysis between oeROR1 and oeCON GSCs. (C) ROR1, CD31, and TJP1 mRNA expression in oeROR1 and oeCON GSC‐1 (*n* = 3). (D) Immunofluorescence staining of TJP1 in oeROR1 and oeCON GSCs. (E) Western blot of relative expression of ROR1, CD31, and TJP1 protein in oeROR1 and oeCON GSCs. (F) The images (left) and (G) quantitative comparison of tube length (right) among shCON, ROR1_sh1, and ROR1_sh2 GSCs in tube formation assay (*n* = 3). (H) GSEA analysis of “ANGIOGENESIS” pathway in differential gene analysis between shROR1 and shCON GSCs. (I) ROR1, CD31, and TJP1 mRNA expression in shROR1 and shCON GSC‐1 (*n* = 3). (J) Immunofluorescence staining of TJP1 in shCON, ROR1_sh1, and ROR1_sh2 GSCs. (K) Western blot of relative expression of ROR1, CD31, and TJP1 protein in shROR1 and shCON GSCs. (L) Dot plot showing expression level of endothelial genes and stemness genes in ROR1− GBM cells and ROR1+ GBM cells from scRNA‐seq of GBM patients. (M) Comparison of GSVA scores for “ANGIOGENESIS” pathway between ROR1− and ROR1+ GBM cells. (N) In vivo bioluminescence imaging of intracranial xenografts at D7 and D18 postimplantation with oeCON, oeROR1, shCON, and shROR1 GSCs, separately. (O) Bar graph comparing in vivo bioluminescence intensity between tumor‐bearing mice implanted with oeCON and oeROR1 GSCs. (P) Comparison of survival between tumor‐bearing mice implanted with oeCON and oeROR1 GSCs. (Q) Bar graph comparing in vivo bioluminescence intensity between tumor‐bearing mice implanted with shCON and shROR1 GSCs. (R) Comparison of survival between tumor‐bearing mice implanted with shCON and shROR1 GSCs. (S) Immunostaining of CD31 and Dextran in xenografts. Bar chart and line chart data are presented as the mean ± SD or mean ± SEM and were analyzed with Student's *t*‐test or one‐way ANOVA. *p < 0.05, **p < 0.01, ***p < 0.001, and ****p < 0.0001 for all figures.

To investigate the role of ROR1 in GDEC formation and function, we overexpressed ROR1 in two GBM‐derived GSCs (Figure S3A). After inducing GDECs from GSCs with or without ROR1 overexpression, we performed in vitro tube formation assays. The results showed that ROR1 overexpression significantly increased the tube length in both GDEC models (*p* = 0.005, Figure 2A and Figure S3B). Gene set enrichment analysis (GSEA) of RNA‐sequencing data from GDECs induced from GSC‐1 revealed that “ANGIOGENESIS” was the only significantly enriched pathway in the ROR1‐overexpression group (adj *p* = 0.048, Figure 2B). Furthermore, RNA‐sequencing data showed a significant increase in ROR1 (*p* < 0.001) and endothelial marker PECAM1 (*p* < 0.001) expression, while adhesion molecule TJP1 expression significantly decreased (*p* < 0.001) in ROR1‐overexpression group (Figure S3C and Data S3).

qPCR experiments further confirmed that ROR1 overexpression upregulated CD31 expression while downregulating TJP1 expression in GDECs derived from both GSCs (Figure 2C and Figure S3D). Immunofluorescence staining and Western blot analysis corroborated these findings, showing increased CD31 protein expression and decreased TJP1 protein expression in the ROR1‐overexpression group compared to the control vector group (Figure 2D,E).

To further validate the role of ROR1 in promoting GDEC angiogenesis, we established ROR1‐knockdown GSC lines (Figure S3E). As expected, in vitro tube formation assays revealed that ROR1 knockdown impaired the vasculogenic capacity of GDECs derived from both GSC lines (Figure 2F,G and Figure S3F). RNA‐sequencing data analysis showed that “ANGIOGENESIS” was significantly not enriched (*p* = 0.016, adj *p* = 0.066, NES = −1.59) in the ROR1‐knockdown group compared to the control group (Figure 2H). Additionally, PECAM1 expression significantly decreased (*p* < 0.001), while TJP1 expression marginally increased (*p =* 0.054) in the ROR1‐knockdown group (Figure S3G and Data S4). qPCR analysis demonstrated that ROR1 knockdown downregulated the PECAM1 while upregulating the TJP1 in GDECs derived from both GSC lines (Figure 2I and Figure S3H). Immunofluorescent staining and Western blot analyses further confirmed that ROR1 knockdown decreased PECAM1 expression while increasing TJP1 expression in GSCs (Figure 2J,K).

After confirming that ROR1 could promote GDEC function in in vitro, we explored its potential role using GBM datasets and scRNA‐seq data. Analysis of bulk RNA‐sequencing data from primary GBM patients in the CGGA database revealed that endothelial genes were more highly expressed (|log2 fold change| > 1, adj. *p* < 0.05) in ROR1‐high GBM samples compared to ROR1‐low samples (Data S5 and Figure S3I). At the single‐cell level, we stratified malignant GBM cells into ROR1+ and ROR1− populations based on the presence or absence of ROR1 expression. ROR1+ cells exhibited significantly higher expression of endothelial markers and stemness genes compared to ROR1− cells (Figure 2L). GSEA analysis further revealed pronounced enrichment of angiogenesis‐related pathways in ROR1+ cells (Figure 2M).

In vivo, orthotopic xenograft models demonstrated that ROR1 overexpression in GSCs accelerated tumor growth (Figure 2N,O and Figure S4A) and resulted in worse survival outcomes (Figure 2P). In contrast, ROR1 knockdown in GSCs reduced tumor growth rates (Figure 2N, Q and Figure S4A) and improved survival compared to controls (Figure 2R). Tumor tissues from the ROR1‐overexpression (oeROR1) group exhibited increased CD133+/CD31+ vascular density (Figure S4B) and decreased TJP1+/CD31+ coexpression area density (Figure S4C) compared to the control vector (oeCON) group. Conversely, the ROR1‐knockdown (shROR1) group showed reduced CD133+/CD31+ vascular density (Figure S4D) and increased TJP1+/CD31+ coexpression area density (Figure S4E) compared to the control (shCON) group.

Importantly, immunofluorescence analysis following tail vein injection of dextran revealed significantly increased dextran leakage around tumor vessels in ROR1‐overexpressing orthotopic tumor models compared to controls (Figure 2S). Conversely, ROR1 knockdown markedly reduced vascular dextran extravasation in the same model (Figure 2S).

Collectively, these findings demonstrate that ROR1 upregulation promotes angiogenesis and enhances the formation and function of GDECs, contributing to increased vascular density in GBM tumors. However, GDEC‐formed vessels exhibit decreased vascular integrity, suggesting that targeting ROR1 may hold potential for promoting vascular normalization in GBM.

### 2.3 WNT5A Serves as an Important ROR1 Ligand Activating WNT/β‐Catenin Signaling in GSCs Which is Mainly Derived from TAMs

**FIGURE 3:**
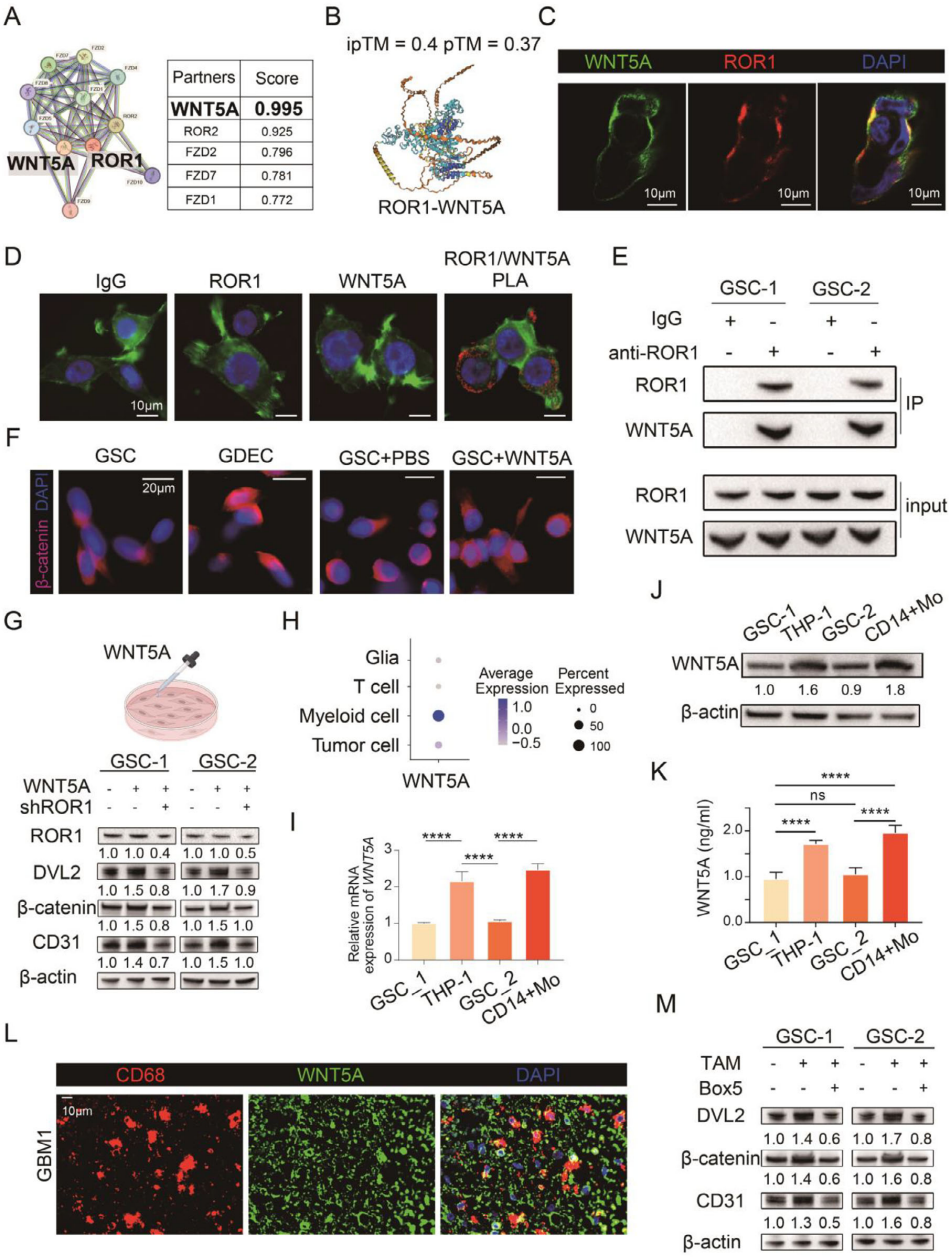
WNT5A serves as the primary ROR1 ligand activating WNT/β‐Catenin signaling in GSCs which is mainly from TAMs. (A) Protein–protein interaction (PPI) network analysis for ROR1. (B) Prediction of ROR1‐WNT5A interactions predicted by AlphaFold3. (C) Immunofluorescence staining of WNT5A, ROR1, and DAPI in GSCs. (D) Proximity Ligation Assay between ROR1 and WNT5A interaction in glioblastoma stem cells by Duolink assay combined with immunofluorescence staining. The red dots indicate their interaction. The β‐actin was labeled as green with phalloidin. (E) Co‐IP analysis in GSC‐1 and GSC‐2. Cell lysates were immunoprecipitated with anti‐ROR1 antibodies followed by immunoblotting with anti‐ROR1 and anti‐WNT5A antibodies. (F) Immunostaining of β‐catenin and DAPI in GSCs under different conditions. (G) Western blot of relative expression of ROR1, DVL2, β‐catenin, CD31, and β‐actin in GSCs under different conditions. (H) Dot plot of scRNA‐seq from 6 GBM patients shows that *WNT5A* is primarily distributed in myeloid cells. (I) WNT5A mRNA expression in GSC‐1, GSC‐2, THP‐1 derived TAMs, and CD14+monocyte derived TAMs. (J) WNT5A protein expression in GSC‐1, GSC‐2, THP‐1 derived TAMs, and CD14+monocyte derived TAMs. (K) The WNT5A protein levels in the supernatant of GSC‐1, GSC‐2, THP‐1 derived glioma‐associated macrophages (GAMs), and CD14+monocyte derived GAMs measured by Enzymelinked immunosorbent assay (*n* = 5). (L) Immunofluorescence staining of CD68, WNT5A, and DAPI in GBM tissue from patients. (M) DVL2, β‐catenin, CD31 protein expression in GSC‐1 and GSC‐2 with or without TAM coculture/TAM coculture combined with Box5. Bar chart data are presented as the mean ± SD or mean ± SEM and were analyzed with Student's *t*‐test or one‐way ANOVA. *p < 0.05, **p < 0.01, ***p < 0.001, and ****p < 0.0001.

Given that ROR1 is a receptor protein, we performed protein–protein interaction (PPI) network analysis and identified WNT5A as the top‐ranking ligand with the strongest binding affinity (Figure 3A). Structural modeling using AlphaFold3, double immunofluorescent staining, proximity ligation assay (PLA), and coimmunoprecipitation (Co‐IP) assays confirmed the colocalization and interaction of WNT5A with ROR1 membrane receptors in GSCs (Figure 3B–E).

Pathway enrichment analysis in protein‐protein‐interaction (PPI) network revealed that ROR1‐associated functions were primarily enriched in the WNT pathway (Figure S5A). To explore whether WNT pathway activation is the downstream effect of WNT5A‐ROR1 binding, we examined the localization of β‐catenin before and after the differentiation of GSCs into GDECs. The results showed a translocation of β‐catenin from the cytoplasm to the nucleus during GDEC differentiation (Figure 3F). Notably, supplementation of WNT5A in GSC culture medium mimicked this nuclear accumulation of β‐catenin (Figure 3F). Furthermore, Western blot analysis demonstrated that ROR1 knockdown suppressed the upregulation of WNT pathway molecules (DVL2 and β‐catenin) and CD31 induced by WNT5A (Figure 3G). qPCR and Western blotting revealed that ROR1 expression in GSCs is not modulated by coculture with TAMs, exogenous WNT5A, or WNT5A antagonist (Box5) (Figure S5B,S5C). These findings suggest that WNT5A‐ROR1 binding promotes GDEC functions by activating the WNT/β‐catenin pathway.

A previous study reported that GSCs in GBM can secrete WNT5A [28]. Here, our scRNA‐seq analysis revealed that WNT5A expression was present in both myeloid cells and GSCs, with predominant expression in myeloid cells (Figure 3H and Figure S5D), which was also confirmed by qPCR, Western blot analyses, enzyme‐linked immunosorbent assay (ELISA), and double immunofluorescence staining for WNT5A and CD68 (Figure 3I–L and Figure S5E). To further investigate the role of TAM‐secreted WNT5A in promoting GDEC formation via interaction with ROR1, we cocultured TAMs with GSCs in the presence or absence of a WNT5A antagonist (Box5). Coculture with TAMs significantly increased CD31 protein expression and activated the Wnt/β‐catenin pathway in GSCs, while treatment with Box5 during coculture effectively suppressed both CD31 expression and Wnt/β‐catenin pathway activation (Figure 3M).

Collectively, these findings demonstrate that TAM‐secreted WNT5A is an important ROR1 ligand that promotes GDEC formation by activating WNT/β‐catenin signaling in GSCs through interaction with ROR1.

### 2.4 M2‐Like TAMs in Hypoxic Microenvironment of GBM as the Main Source of WNT5A

**FIGURE 4:**
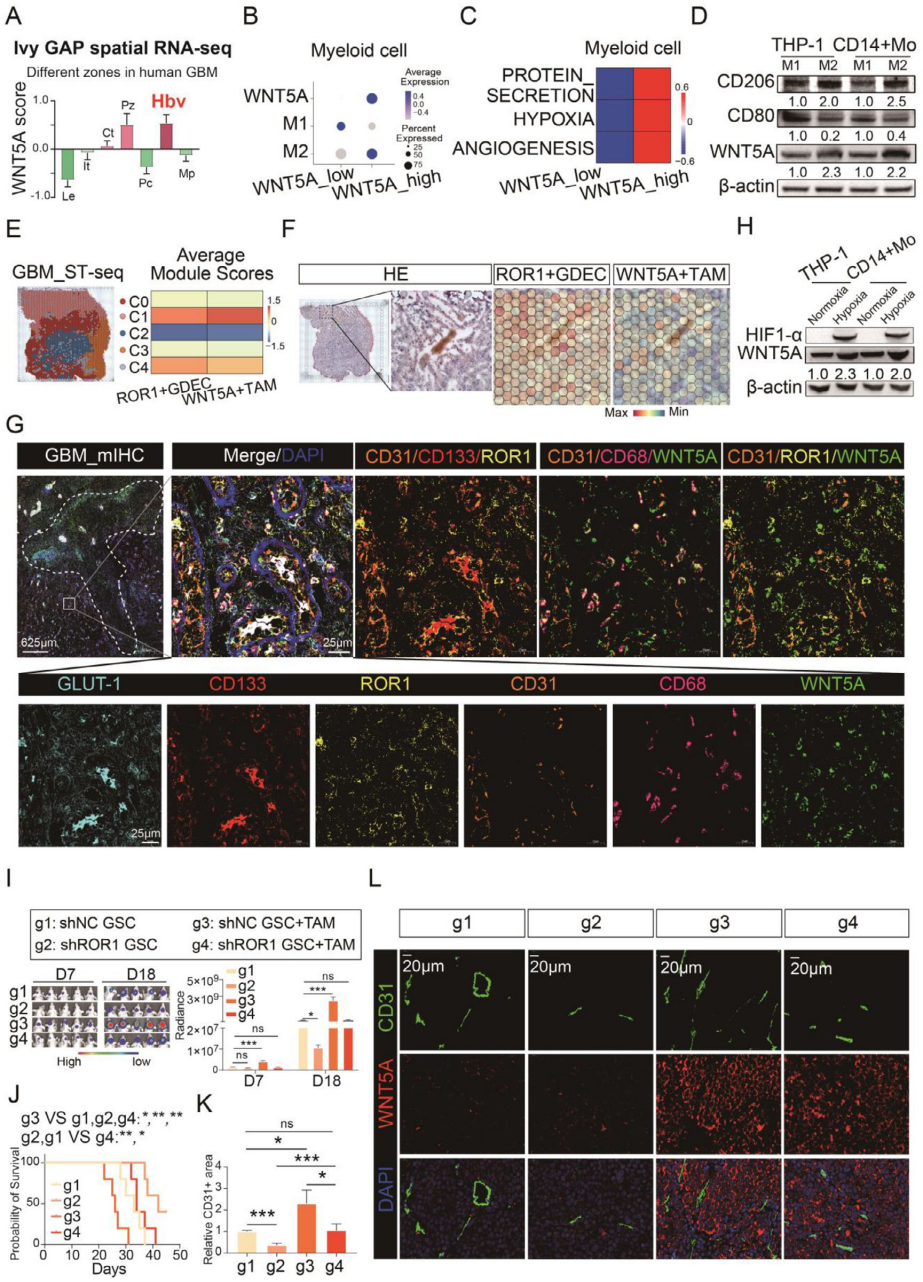
WNT5A is predominantly secreted by M2‐like TAM in the hypoxic microenvironment and activates WNT5A‐ROR1‐WNT pathway in GSC to promote GDEC differentiation. (A) Bar chart of expression level of WNT5A in Ivy GAP spatial RNA‐seq from GBM patients. (B) Dot plot of *WNT5A*, *M1*, *M2* genes expression in WNT5A_high and WNT5A_low TAMs grouped by scRNA‐seq from 6 GBM patients. (C) Heatmap of GSVA scores for “PROTEIN_SECRETION,” “HYPOXIA,” and “ANGIOGENESIS” in WNT5A_high and WNT5A_low TAMs. (D) CD206, CD80, WNT5A protein expression in M1‐phenotype and M2‐phenotye TAMs derived from THP‐1 or CD14+monocyte. (E) Spatial transcriptome sequencing (ST‐seq) of GBM tissue from 1 patient and the sequencing spot were clustered into 5 clusters (C0–C4) after dimensionality reduction (left). The average module scores of expression level of marker genes of ROR1+GDEC and WNT5A+TAM in 5 spatial clusters (right). (F) Adjacent H&E‐stained section of GBM tissue for spatial transcriptomics, with magnified views of necrotic and angiogenic regions (left). Heat map of spatial expression distribution of marker genes of ROR1+GDEC and WNT5A+TAM (right). (G) Multiplex immunohistochemistry of tissue section from GBM patient for GLUT‐1, CD133, ROR1, CD31, CD68, and WNT5A. The hypoxic necrotic zone is demarcated by white dotted lines. (H) Western blot of relative expression of HIF1‐α and WNT5A in THP‐1/CD14+monocyte‐derived TAM under normoxia or hypoxia condition. (I) Representative bioluminescent images of mice bearing tumor from 4 groups (left). The luminescence signal intensity of intracranial tumor in 4 groups (right). (J) Kaplan−Meier survival curve of mice bearing intracranial tumor (*n* = 5 per group). (K) Relative CD31 expression density across four tumor‐bearing mice groups (*n* = 3). (L) Immunofluorescence staining of CD31, WNT5A, and DAPI in GBM tissue from tumor‐bearing mice in 4 groups. Bar chart data are presented as the mean ± SD or mean ± SEM and were analyzed with Student's *t*‐test or one‐way ANOVA. *p < 0.05, **p < 0.01, ***p < 0.001, and ****p < 0.0001 for all figures.

In the TCGA GBM RNA‐sequencing data, we found significantly higher WNT5A expression in M2‐like macrophages compared to M0‐like and M1‐like macrophages (*p* < 1e‐15, Figure S6A). Further, both WNT5A and the M2 macrophage marker CD163 were highly expressed in the Hbv regions, aligning with the spatial distribution of ROR1 expression (Figure 4A and Figure S6B). After stratifying myeloid cells from scRNA‐seq of GBM samples into WNT5A_High (> 0.5) and WNT5A_Low (≤ 0.5) populations (Figure S6C,S6D), we found that WNT5A_High myeloid cells exhibited a predominantly M2‐like polarization state and enrichment of pathways associated with “PROTEIN_SECRETION,” “ANGIOGENESIS,” and “HYPOXIA” (Figure 4B,C), suggesting that WNT5A_High myeloid cells may promote angiogenesis through secretory mechanisms under hypoxic conditions. We polarized THP‐1 cells and CD14+ monocytes into M1‐like and M2‐like macrophages and confirmed that M2‐polarized macrophages exhibited significantly higher WNT5A expression compared to M1‐polarized macrophages at both the mRNA and protein levels (Figure 4D and Figure S6E).

Using 10x Visium spatial transcriptomics on GBM patient specimens, we identified five distinct clusters through dimensionality reduction and clustering (Figure 4E and Figure S7A). ROR1+ GDECs and WNT5A+ TAMs exhibited concordant spatial distribution patterns in both our sequencing data and previously published data (GSE194329) (Figure 4E and Figure S7B–S7E). Within the microvascular niche, ROR1+ GDECs and WNT5A+ TAMs showed higher signals compared to adjacent regions (Figure 4F). Given that ROR1+/CD31+ GDECs were primarily observed in hypoxic regions of GBM samples (Figure 1K), we performed costaining of GLUT‐1, CD133, ROR1, CD31, CD68, and WNT5A in a consecutive tissue section adjacent to Figure 1K. In the hypoxic region surrounding the necrotic area (white dotted line), we observed a prominent infiltration of WNT5A+TAMs (coexpression of CD68 and WNT5A) surrounding ROR1+GDECs (coexpression of ROR1, CD133, and CD31) (Figure 4G). Notably, secreted WNT5A was observed binding to ROR1 on ROR1+ GDECs (coexpression of CD31, ROR1, and WNT5A) (Figure 4G). Western blot analysis further confirmed that hypoxia induced upregulation of WNT5A expression in TAMs (Figure 4H). ELISA results confirmed that hypoxia elevates WNT5A protein secretion in the coculture system (Figure S7F).

In vivo, orthotopic tumor models demonstrated that coinjection GSCs with myeloid cells markedly accelerated tumor growth. However, this pro‐tumorigenic effect was significantly attenuated when myeloid cells were coinjected with ROR1‐knockdown GSCs (Figure 4I and Figure S7G). Survival analysis revealed that mice receiving coinjected GSCs and myeloid cells had the shortest overall survival, while those injected with ROR1‐knockdown GSCs showed the longest survival (Figure 4J). Mice injected with control GSCs or with ROR1‐knockdown GSCs and myeloid cells exhibited comparable survival durations. Fluorescence staining of tumor tissue sections revealed that the coinjection of myeloid cells with GSCs resulted in the highest tumor vascular density, while GSCs with ROR1‐knockdown alone showed the lowest tumor vascular density (Figure 4K and Figure S7H). The control GSC group and the coinjection group of myeloid cells with ROR1‐knockdown GSCs displayed comparable tumor vascular densities (Figure 4K and Figure S7H). Furthermore, CD31/WNT5A costaining confirmed significantly higher perivascular WNT5A expression in tumors derived from the co‐injection of myeloid cells and GSCs compared to tumors formed by GSCs alone (Figure 4L).

Together, these findings indicate that M2‐like TAMs in the hypoxic microenvironment of GBM are the primary source of WNT5A and play a critical role in promoting GBM progression by enhancing angiogenesis through the interaction between WNT5A and ROR1.

### 2.5 Hypoxia‐Driven WTAP Increases ROR1 Stability in GSCs Through m^6^A Modification in an HuR‐Dependent Manner

**FIGURE 5:**
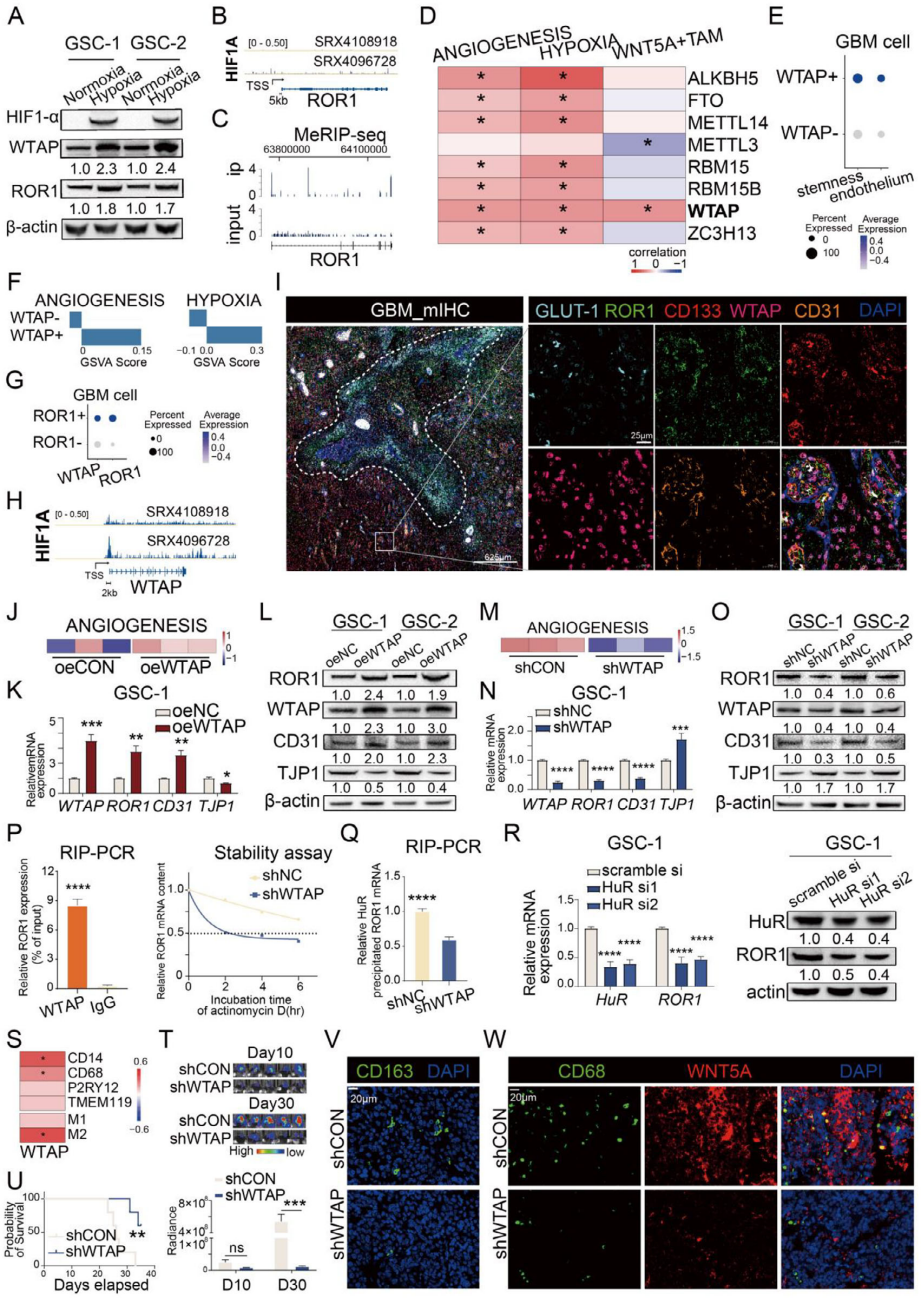
Hypoxia‐driven WTAP increases ROR1 stability in GSCs to promote GDEC differentiation through m^6^A modification in an HuR‐dependent manner. (A) Western blot of relative expression of HIF1‐α, WTAP, and ROR1 in two GSC lines under normoxia or hypoxia condition. (B) ChIP‐seq analysis of HIF1‐α in tumor cells (LNCAP and PC‐3) and the representative Integrative Genomics Viewer (IGV) tracks at the ROR1 locus. (C) MeRIP‐sequencing data of ROR1 in GSCs. (D) Correlation analysis between methyltransferases/demethylases and the gene sets (“ANGIOGENESIS,” “HYPOXIA,” “WNT5A+TAM”) in GBM RNA‐seq data. (E) Dot plot showing expression level of endothelial genes and stemness genes in WTAP+ GBM cells and WTAP‐ GBM cells from scRNA‐seq of 6 GBM patients. (F) Comparison of GSVA scores for “ANGIOGENESIS” (top) and “HYPOXIA” pathway (bottom) between WTAP+ and WTAP‐ GBM cells. (G) Dot plot showing expression level of *WTAP* and *ROR1* in ROR1+ GBM cells and ROR1− GBM cells from scRNA‐seq of 6 GBM patients. (H) ChIP‐seq analysis of HIF1‐α in tumor cells (LNCAP and PC‐3) and the representative Integrative Genomics Viewer (IGV) tracks at the WTAP locus show the distribution of peaks upstream of TSS. (I) Multiplex immunohistochemistry of tissue section from GBM patient for GLUT‐1, ROR1, CD133, CD31, WTAP, and DAPI. The hypoxic necrotic zone is demarcated by white dotted line. (J) GSVA analysis of “ANGIOGENESIS” pathway in differential gene analysis between oeWTAP and oeCON GSCs. (K) WTAP, ROR1, CD31, TJP1 mRNA expression in oeWTAP and oeNC GSC‐1 (*n* = 3). (L) WTAP, ROR1, CD31, and TJP1 protein expression in oeWTAP and oeNC GSCs. (M) GSVA analysis of “ANGIOGENESIS” pathway in differential gene analysis between shWTAP and shCON GSCs. (N) WTAP, ROR1, CD31, TJP1 mRNA expression in shWTAP and shNC GSC‐1 (*n* = 3). (O) WTAP, ROR1, CD31, and TJP1 protein expression in oeWTAP and oeNC GSCs. (P) RIP‐PCR showing the content of *ROR1* mRNA immunoprecipitated by WTAP antibody. IgG antibodies were used as negative control (left). The stability of ROR1 mRNA in GSCs with or without WTAP knockdown (right). (Q) RIP‐qPCR showing the content of *ROR1* immunoprecipitated by HuR antibody in GSCs with or without WTAP shRNA (*n* = 3). (R) HuR and ROR1 mRNA (left) and protein (right) expression in scramble si, HuR si1, HuR si2 GSC‐1 (*n* = 3). (S) Correlation analysis between WTAP and marker genes of TAM (CD14, CD68, P2RY12, TMEM119, M1 genes, M2 genes) in GBM RNA‐seq data. (T) Representative bioluminescent images of C57 mice bearing gl261‐derived tumor from 2 groups (top). The luminescence signal intensity of intracranial tumor in 2 groups (bottom) (*n* = 5). (U) Kaplan−Meier survival curve of mice bearing intracranial tumor (*n* = 5 per group). Immunofluorescence staining of CD163 and DAPI (V), CD68, WNT5A, and DAPI (W) in GBM tissue from tumor‐bearing mice in 2 groups. Bar chart and line chart data are presented as the mean ± SD or mean ± SEM and were analyzed with Student's *t*‐test or one‐way ANOVA. *p < 0.05, **p < 0.01, ***p < 0.001, and ****p < 0.0001 for all figures.

By utilizing qPCR, western blotting, and in vitro tube formation, we verified that hypoxic coculture conditions could promote the endothelial differentiation of GSCs (Figure S8A–S8C). Although, Western blot result demonstrated that hypoxia upregulated ROR1 expression (Figure 5A), ChIP‐seq datasets showed that HIF1‐α did not bind to the ROR1 promoter (Figure 5B). These findings suggest that hypoxia may regulate ROR1 expression through alternative mechanisms. We and others have reported that RNA N6‐methyladenosine (m^6^A) plays a vital role in promoting glioma malignant progression and tumor angiogenesis [29, 30, 31, 32]. Here, we performed Methylated RNA Immunoprecipitation Sequencing (MeRIP‐seq) and found that ROR1 can be modulated by m^6^A modification in GSCs (Figure 5C), To explore potential m^6^A‐associated upstream regulators of ROR1, we analyzed the correlation between methyltransferases/demethylases and the gene sets for “ANGIOGENESIS,” “HYPOXIA,” and “WNT5A+TAM” in TCGA GBM RNA‐seq data, revealing that only WTAP exhibited a positive correlation with all three gene sets (Figure 5D). WTAP expression was increased with tumor grade (Figure S9A–S9D) and positively correlated with ROR1 in CGGA GBM dataset (Figure S9E). In scRNA‐seq data, WTAP‐positive‐expression (WTAP+) GBM cells exhibited significantly higher expression of stemness‐related and endothelial gene sets compared to WTAP‐negative‐expression (WTAP−) GBM cells (Figure 5E). Enrichment analysis revealed that WTAP+ GBM cells displayed markedly upregulated pathway activity in “ANGIOGENESIS” and “HYPOXIA” compared to WTAP− GBM cells (Figure 5F). Meanwhile, scRNA‐seq data also showed significantly higher WTAP expression in ROR1+ GBM cells compared to ROR1− GBM cells (Figure 5G).

Moreover, ChIP‐seq data analysis revealed that HIF1‐α binds to the promoter region of WTAP, thus driving its transcription (Figure 5H). Western blot results showed elevated protein expression of WTAP under hypoxic conditions (Figure 5A), suggesting that hypoxia may regulate ROR1 expression through WTAP expression. Multiplex staining of GLUT‐1, CD133, ROR1, WTAP, and CD31 using GBM slices adjacent to those used in Figure 1A showed colocalization of WTAP, ROR1, CD133, and CD31 in the hypoxic regions surrounding the same necrotic area (white dotted line) (Figure 5I). These findings indicate that WTAP may serve as an upstream regulator of ROR1 through m^6^A modification under hypoxic conditions.

To directly investigate the impact of WTAP expression on ROR1 expression, we constructed WTAP‐overexpression GSCs (Figure S9F). RNA‐seq analysis revealed that WTAP overexpression significantly upregulated WTAP, ROR1, and PECAM1 expression while downregulating the adhesion molecule TJP1 (Figure S9G and Data S6). Furthermore, enrichment analysis demonstrated enhanced activity of both the “ANGIOGENESIS” pathway and GDEC gene set following WTAP overexpression (Figure 5J and Figure S9H). qPCR and Western blot showed that WTAP overexpression increased mRNA and protein expression of ROR1 and PECAM1 and decreased protein levels of TJP1 (Figure 5K,L and Figure S9I,S9K). We also established WTAP‐knockdown GSC cells (Figure S9L). RNA‐seq analysis revealed that WTAP knockdown markedly decreased WTAP, ROR1, and PECAM1 levels, while upregulating TJP1 expression (Figure S9M and Data S7). Additionally, enrichment analysis demonstrated reduced activity of both the “ANGIOGENESIS” pathway and GDEC gene set following WTAP knockdown (Figure 5M and Figure S9N). qPCR and Western blot confirmed that WTAP knockdown downregulated WTAP, ROR1, and PECAM1 levels while upregulating TJP1 expression (Figure 5N, O and Figure S9O–S9Q). Moreover, RIP‐PCR revealed that WTAP binds to ROR1 mRNA (Figure 5P). mRNA stability assays showed that WTAP knockdown decreased the mRNA stability of ROR1 (Figure 5P). Next, RIP‐qPCR demonstrated that the m^6^A reader protein HuR binds to ROR1 mRNA, with this binding significantly reduced following WTAP knockdown (Figure 5Q). qPCR and Western blot showed that HuR knockdown downregulated ROR1 expression (Figure 5R and Figure S9R). These findings indicate that WTAP stabilizes ROR1 in GSCs through m^6^A modification in an HuR‐dependent manner.

It has been reported that WTAP upregulation can promote macrophage infiltration in gliomas [33]. In RNA‐seq data of GBM patients, we confirmed a positive correlation between WTAP and M2 macrophage infiltration (Figure 5S and Figure S10A). To simultaneously investigate the role of WTAP in regulating ROR1 and macrophage infiltration in vivo, we utilized WTAP‐knockdown GL261 cells to establish orthotopic tumor models (Figure S10B). We observed significantly slower tumor growth and prolonged survival in mice bearing WTAP‐knockdown tumors (Figure 5T,U and Figure S10C). In the tumor tissue formed by GL261 with WTAP knockdown, we noted decreased WTAP and CD31 expression along with reduced M2 macrophage infiltration (Figure 5V and Figure S10D–S10F). Furthermore, these tumors exhibited reduced recruitment of WNT5A+ TAMs (Figure 5W and Figure S10F,S10G), which may also account for the diminished abnormal vascular proliferation and delayed tumor progression.

Together, these findings indicate that upregulated WTAP increases ROR1 stability in GSCs through m^6^A modification in an HuR‐dependent manner within the hypoxic microenvironment. Concurrently, upregulated WTAP has the potential to enhance WNT5A‐ROR1 interactions by promoting the infiltration of TAMs.

### 2.6 Targeting the ROR1‐WNT5A Axis Promotes Vascular Normalization of Tumor and Enhances TMZ Efficacy in GBM

**FIGURE 6:**
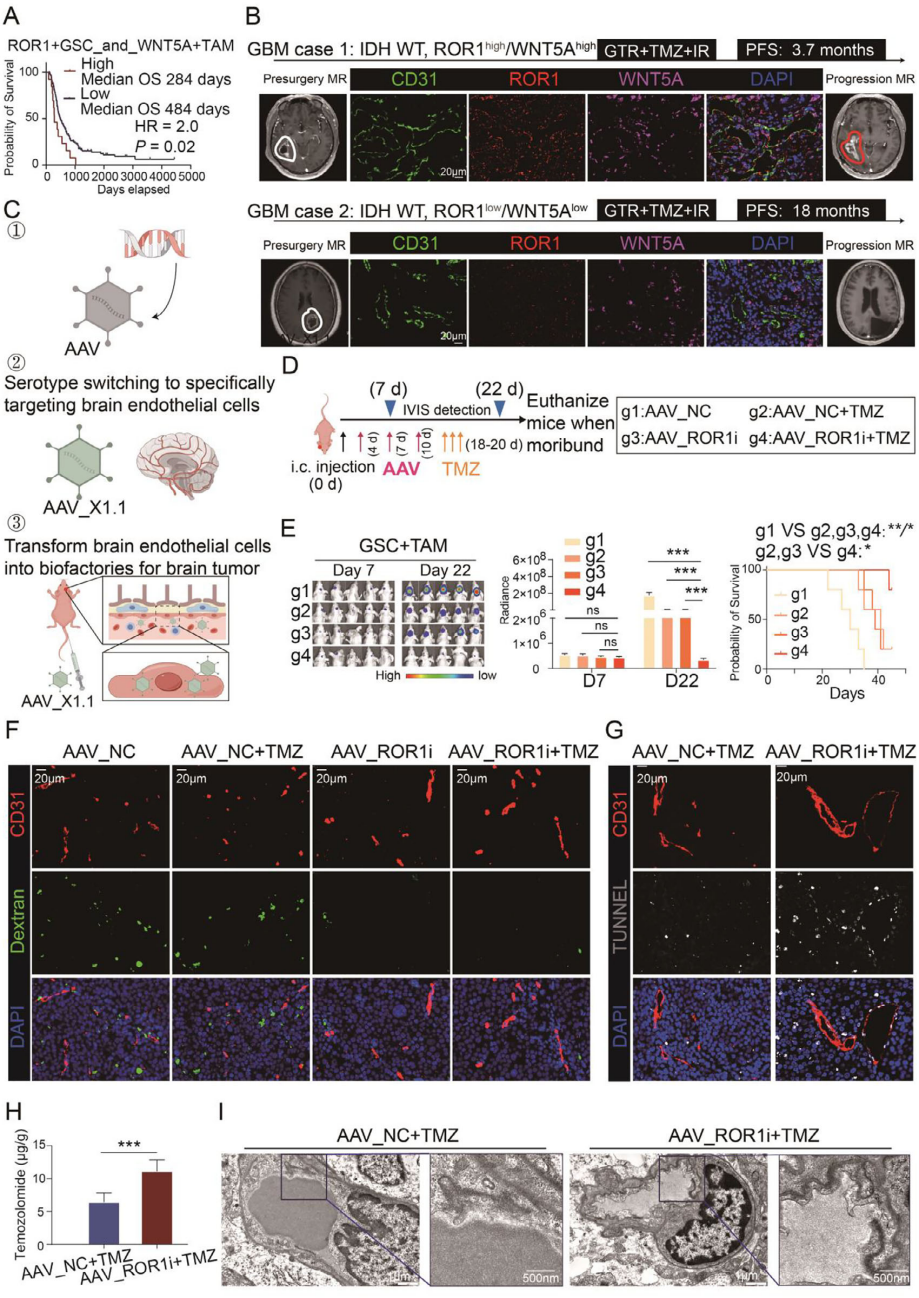
Clinical significance and therapeutic targeting potential of the ROR1‐WNT5A axis in glioblastoma. (A) Kaplan−Meier survival curve of primary GBM patients with standard treatment grouped by high or low expression of ROR1+GSC and WNT5A+TAM. (B) Therapeutic response of TMZ in two representative GBM patients with high or low CD31/ROR1/WNT5A expression. Representative MRI, immunofluorescence staining of CD31, ROR1, and WNT5A and PFS were shown. Tumor border was marked by white line in presurgery MRI and red line in progression MRI. Patients with low CD31/ROR1/WNT5A benefit more from TMZ treatment relative to those with high CD31/ROR1/WNT5A. (C) Schematic diagram of AAV X1.1 specifically targeting endothelial cells. (D) Schematic diagram of the combined treatment of AAV and TMZ in tumor‐bearing mice. Mice bearing tumors were treated with 3 intravenous (i.v.) injections of AAV_ROR1‐shRNA (AAV_ROR1i) or AAV_NC (dose range: 5 × 10^11^ to 1 × 10^12^ vg/injection in 100 mL DPBS) at Days 4, 7, and 10 after tumor implantation. And TMZ (5 mg/kg, i.p.) was given for 3 consecutive days since Day 18 after tumor implantation. The growth of tumor was monitored by bioluminescence imaging on Days 7 and 22 after coinjection of GSCs and THP‐1 cells. (E) Representative bioluminescent images of mice bearing tumor from 4 groups (left). The luminescence signal intensity of intracranial tumor in 4 groups (middle). Kaplan−Meier survival curve of mice bearing intracranial tumor (*n* = 5 per group) (right) (*n* = 5). (F) Immunofluorescence staining of CD31, Dextran, and DAPI in GBM tissue from tumor‐bearing mice in 4 groups. (G) Immunofluorescence staining of CD31, TUNNEL, and DAPI in GBM tissue from tumor‐bearing mice in two groups with TMZ treatment. (H) Quantification of TMZ concentration in tumor from tumor‐bearing mice in two groups with TMZ treatment (*n* = 5). (I) TEM images of endothelial tight junction in tumor from tumor‐bearing mice in two groups with TMZ treatment. Bar chart and line chart data are presented as the mean ± SD or mean ± SEM and were analyzed with Student's *t*‐test or one‐way ANOVA. *p < 0.05, **p < 0.01, ***p < 0.001, and ****p < 0.0001 for all figures.

To characterize the prognosis or therapeutic role of ROR1‐WNT5A axis in GBM, we collected expression levels of these genes in GBM patients receiving standard treatments within the CGGA dataset. The results indicated that patients with tumors exhibiting high expression of the gene set “ROR1, CD133, WNT5A, and CD68” had significantly shorter overall survival compared to those with low expression of this gene set (Figure 6A). Consistently, in two representative cases of IDH‐wildtype GBM with complete tumor resection and standard Stupp treatment regimen, the case with high ROR1 and WNT5A expression in CD31+ endothelial cells experienced a rapid relapse 3.8 months after treatment (Figure 6B). In contrast, the case with low ROR1 and WNT5A expression showed no relapse for 18 months. These findings suggest that ROR1 and WNT5A expression may serve as potential predictors of TMZ efficacy in gliomas.

Notably, when analyzing differentially expressed genes in extracellular vesicles from blood samples of six GBM patients and six healthy controls in the GSE106804 dataset, we found that WNT5A expression was elevated with borderline statistical significance (log2 FC = 4.76, adj *p* = 0.05) in the vesicles of GBM patients (Data S8 and Figure S11A). Additionally, we newly collected 10 peripheral blood samples from GBM patients, low‐grade glioma (LGG) patients, and five healthy volunteers for ELISA analysis of WNT5A levels. The results demonstrated that WNT5A concentrations were significantly higher in LGG patients (*p* = 0.0003) compared to healthy controls, and further elevated (*p* < 0.0001) in GBM patients relative to LGG cases (Figure S11B). These findings indicate that blood levels of WNT5A could serve as a circulating biomarker for higher‐grade gliomas.

scRNA‐seq analysis of endothelial cells from three GBM cases and paired peritumoral endothelial cells revealed ROR1 specifically expressed in tumor endothelial cells (Figure S11C–S11J). Thus, the critical role of ROR1 in promoting glioma progression also provides a potential therapeutic opportunity. A recent study reported an endothelial‐specific AAV infection system [34]. Here, we also employed endothelial‐specific AAV_X1.1‐mediated ROR1 knockdown in combination with TMZ to treat orthotopic tumor‐bearing mice (Figure 6C,D). The combination therapy significantly controlled tumor progression and improved survival (Figure 6E and Figure S11K). Fluorescein leakage experiment revealed that AAV_ROR1‐shRNA (AAV_ROR1i) induced tumor vessel normalization and reduced vascular leakage (Figure 6F). TUNEL assays demonstrated enhanced TMZ cytotoxicity around tumor vessels when combined with AAV_ROR1i (Figure 6G). High‐performance liquid chromatograph (HPLC) analysis indicated higher intratumoral TMZ concentrations in the combination group compared to TMZ monotherapy group (*p* = 0.0005, Figure 6H and Figure S11L). Transmission electron microscopy further showed that AAV_ROR1i increased tight junction density between tumor vascular endothelial cells (Figure 6I). Together, these findings revealed that targeting ROR1 promotes vascular normalization of tumor and enhances TMZ efficacy in GBM.

### 2.7 A Novel ROR1‐Targeting Antibody‐Drug Conjugate, VLS‐101, Shows Promising Clinical Applicability in GBM Therapy

**FIGURE 7:**
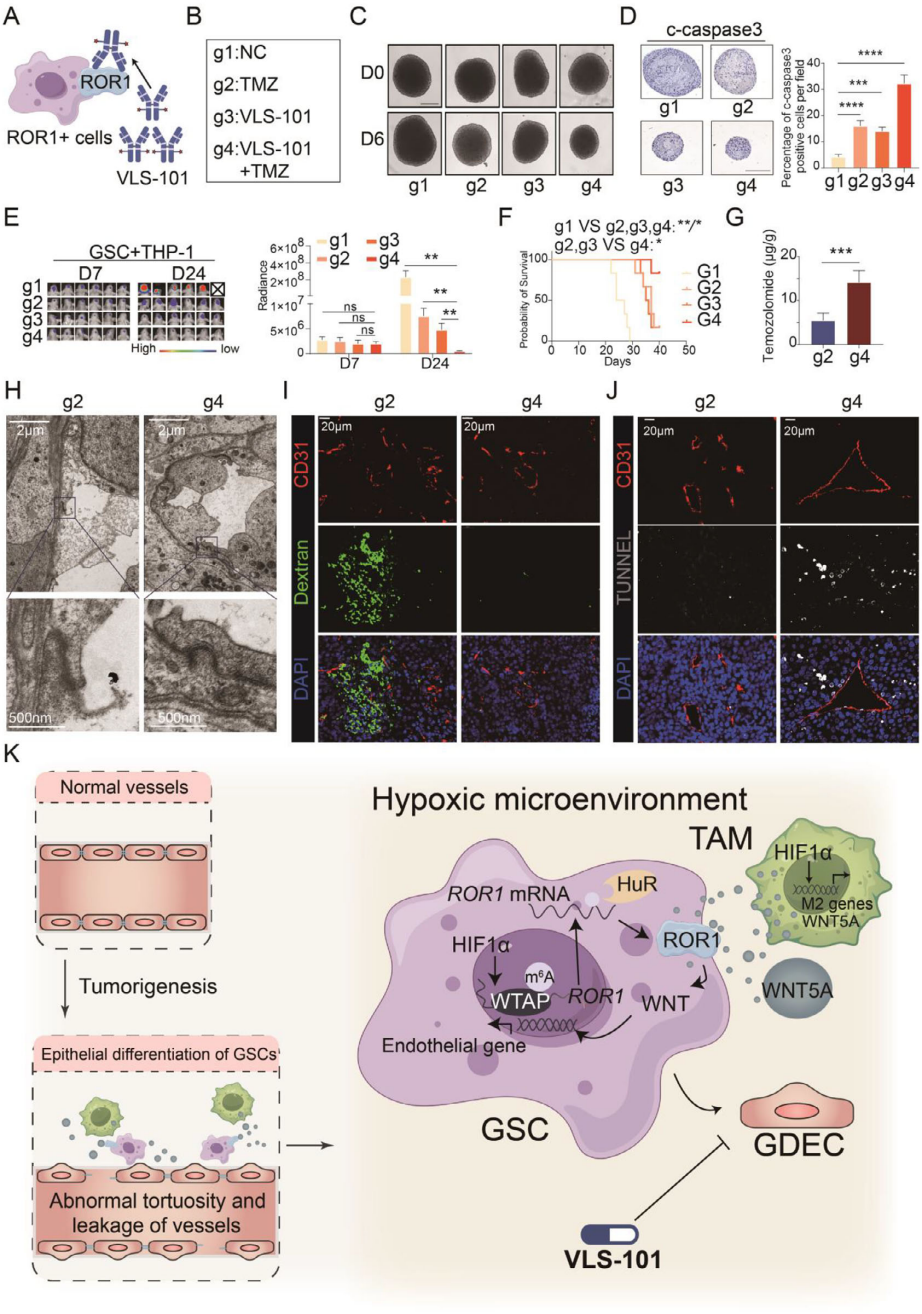
The antibody drug conjugate VLS‐101 targeting ROR1 is effective in GBM therapy and shows promising clinical applicability. (A) Schematic diagram of VLS‐101 targeting ROR1+ GSCs. (B) Four experimental groups based on combination therapy of VLS‐101 and TMZ. (C) Response of GBM patient‐derived organoids to different therapeutic combinations for 6 days. Scale bars, 200 µm. (D) Expression of c‐caspase3 in organoids after different therapeutic combinations for 6 days by IHC. Scale bars, 200 µm. (E) Representative bioluminescent images of mice bearing tumor from 4 groups receiving different combination therapy of VLS‐101 and TMZ (left). The luminescence signal intensity of intracranial tumor in 4 groups (right) (*n* = 6). (F) Kaplan−Meier survival curve of mice bearing intracranial tumor (*n* = 6 per group). (G) Quantification of TMZ concentration in tumor from tumor‐bearing mice in two groups with TMZ treatment (*n* = 6). (H) TEM images of endothelial tight junction in tumor from tumor‐bearing mice in two groups with TMZ treatment. (I) Immunofluorescence staining of CD31, Dextran, and DAPI in GBM tissue from tumor‐bearing mice in two groups with TMZ treatment. (J) Immunofluorescence staining of CD31, TUNEL, and DAPI in GBM tissue from tumor‐bearing mice in two groups with TMZ treatment. (K) A schematic model of TAM‐GSC crosstalk in promoting GDEC differentiation. In hypoxic microenvironment, WNT5A is predominantly secreted by TAMs, with minor autocrine production by GSCs. Hypoxia‐induced WTAP mediates ROR1 stability by m^6^A modifications in a HuR‐dependent manner in GSC. WNT5A in microenvironment activates the WNT pathway via ROR1 binding on GSCs, driving GDEC differentiation. Therapeutic targeting of ROR1 with VLS‐101 inhibits GDEC formation, promotes vascular normalization, and enhances TMZ efficacy. Bar chart and line chart data are presented as the mean ± SD or mean ± SEM and were analyzed with Student's *t*‐test or one‐way ANOVA. *p < 0.05, **p < 0.01, ***p < 0.001, and ****p < 0.0001 for all figures.

The ROR1‐targeting antibody‐drug conjugate (ADC) agent VLS‐101 has demonstrated promising therapeutic efficacy in Mantle cell lymphoma (Figure 7A) [35]. The specific upregulation of ROR1 in GBM also presents a therapeutic opportunity for VLS‐101 in this tumor. Therefore, we evaluated the combination of VLS‐101 with temozolomide in GBM‐derived organoids and orthotopic glioma mouse models to determine their synergistic effects (Figure 7B). Organoid models revealed that VLS‐101 and TMZ combination therapy exhibited superior tumor‐killing efficacy compared to monotherapies (Figure 7C,D). In orthotopic glioma mouse models, the combination therapy significantly controlled tumor progression and improved survival (Figure 7E,F and Figure S12A). HPLC analysis revealed higher intratumoral TMZ concentrations in the combination group compared to TMZ monotherapy (*p* = 0.0006, Figure 7G and Figure S12B). Transmission electron microscopy further showed that VLS‐101 increased tight junction density between tumor vascular endothelial cells (Figure 7H). The fluorescein leakage test confirmed that VLS‐101 induced tumor vessel normalization and reduced vascular leakage (Figure 7I and Figure S12C). TUNEL assays demonstrated enhanced TMZ cytotoxicity around tumor vessels when combined with VLS‐101 (Figure 7J). These findings underscore the promising therapeutic effects of the ROR1‐targeting ADC VLS‐101 in the treatment of GBM.

## 3 Discussion

Hypoxia is a defining hallmark of GBM, driven by a structurally and functionally abnormal vasculature characterized by hyperpermeability and poor perfusion [9]. This vascular instability not only exacerbates the hypoxic niche by limiting oxygen supply but also compromises drug delivery to the tumor parenchyma, thereby contributing to therapeutic resistance [15, 36]. A growing body of evidence indicates that GSCs can differentiate into functional endothelial cells, participate in vasculogenic mimicry, and contribute to tumor vascularization, thereby facilitating glioma invasion and drug resistance [37, 38, 39]. Despite these advances, the role of hypoxia in regulating the differentiation of GSCs into GDEC within GBM microenvironment remains incompletely understood.

Here, we address this knowledge gap by demonstrating that upregulated interaction between ROR1 and WNT5A plays a crucial role in the GDEC formation under hypoxic conditions (Figure 7K). Mechanistically, HIF1‐α drives the transcription of WTAP in GSCs, where WTAP stabilizes ROR1 mRNA through m^6^A modification in an HuR‐dependent manner, leading to increased ROR1 expression. Concurrently, WTAP upregulation mediates the increased infiltration of TAMs. The WNT5A secreted mainly by the hypoxia‐driven TAMs, can bind to ROR1, activating the WNT pathway to induce endothelial‐like gene transcription, thereby promoting GSC differentiation into GDECs. Importantly, we also demonstrated that both endothelial‐specific AAV‐mediated ROR1 knockdown and antibody drug conjugate VLS‐101 can inhibit GDECs formation, improving glioma vessel integrity, enhancing TMZ delivery, and increasing antitumor efficacy. These findings hold significant promise for clinical application.

ROR1 is a transmembrane protein belonging to the receptor tyrosine kinase (RTK) family, comprising both extracellular and intracellular domains. In various types of tumors, ROR1 demonstrates elevated expression while maintaining low expression levels in normal tissues [40]. In GBM, ROR1 has been proved its preferentially expressed in GSCs [41], while its pro‐angiogenic function remains unverified. Here, we demonstrated that ROR1 exhibited significantly elevated expression specifically in GBM vessels, with negligible detection in peritumoral blood vessels. Functional studies further demonstrated that ROR1 is a critical regulator of GSC‐to‐GDEC differentiation, acting through the activation of WNT signaling. This finding uncovers a novel mechanism of tumor angiogenesis under hypoxia, complementing existing knowledge that WNT signaling modulates BBB formation and function in GBM, as well as phenotypic switching of vasculature following therapy processes that directly influence drug delivery and tumor recurrence [42, 43]. Specifically, our data showed that the WNT5A‐ROR1 interaction in GBM activates WNT signaling, thereby promoting GDEC formation and contributing to the development of aberrant and hyperpermeable tumor vasculature.

While the detailed downstream molecular cascades through which ROR1‐WNT signaling drives angiogenesis require further investigation, we observed a key therapeutic advantage: pharmacological targeting of ROR1 stabilizes endothelial junctions in tumor vessels without affecting the integrity of normal brain vasculature. Together, these results position ROR1 as both a poor prognostic biomarker and a promising therapeutic target in GBM.

Tumor vasculature is an integral component of the GBM microenvironment, and its structural and functional abnormalities have broad consequences: they impede the infiltration of antitumor immune cells, fostering an immunosuppressive niche, while also interacting dynamically with microenvironmental cells to drive angiogenesis [27, 44, 45]. TAMs, the most abundant immune cells in GBM, exhibit marked phenotypic plasticity, enabling them to interact with tumor cells, T cells, and endothelial cells to promote malignancy through immune evasion and tumor progression [9, 18, 46]. Under hypoxia, TAMs are recruited to the tumor niche, undergo polarization toward an immunosuppressive (M2‐like) phenotype, and contribute to angiogenesis [9, 17]. Despite this recognition, the direct role of TAMs in regulating GDEC formation remained poorly characterized. Using single‐cell multiomics analysis, we identified TAMs as the primary source of WNT5A in GBM. Functional validation further revealed that crosstalk between WNT5A^+^ TAMs and ROR1^+^ GSCs activates WNT signaling to drive GDEC differentiation, with hypoxia‐induced WNT5A secretion from TAMs directly stimulating this process. These findings illuminate the interconnected interplay between tumor cells (GSCs), immune cells (TAMs), and angiogenesis within the hypoxic GBM niche, and provide insight into the dynamic evolution of glioma progression.

Among post‐transcriptional RNA modifications, m^6^A methylation is the most extensively studied and functionally diverse [47, 48]. RNA m^6^A is a dynamic and reversible modification regulated by methyltransferases (“writers”), demethylases (“erasers”), and m^6^A‐binding proteins (“readers”). WTAP, a core component of the m^6^A methyltransferase complex, has been demonstrated to promote the pathogenesis and progression of various diseases through RNA stabilization [6, 49]. In GBM, WTAP was previously reported to drive the formation of an immunosuppressive microenvironment [33]. Here, our study extends this finding by demonstrating two complementary roles of WTAP in GBM angiogenesis: first, WTAP‐mediated TAM infiltration contributes to angiogenesis by enhancing WNT5A secretion; second, WTAP upregulates the expression of ROR1 (the WNT5A receptor) by stabilizing ROR1 mRNA via HuR‐dependent m^6^A modification. Together, these dual actions of WTAP converge to promote aberrant vasculogenesis in GBM.

The heterogeneous BTB in glioma is a major barrier to effective therapy, as it results in regional variability in drug delivery and ultimately compromises therapeutic efficacy [50, 51]. Current strategies to enhance drug penetration in GBM include vascular normalization, administration of the anti‐VEGF antibody bevacizumab, transient BBB disruption using focused ultrasound, and directly delivery drugs into cerebral‐spinal fluid [9, 52, 53, 54, 55]. However, these approaches have limitations: bevacizumab is associated with acquired resistance and adverse vascular events, while focused ultrasound carries risks of cerebral edema, hemorrhage, and elevated interstitial pressure, which can further impede tumor perfusion [53]. Our work identified that WNT5A/ROR1 interaction as a promising target for GBM vascular normalization, thereby promoting drug delivery efficiency. In orthotopic GBM mouse models, treatment with VLS‐101 (the ROR1‐targeting ADC) induced vascular normalization, enhanced intratumoral TMZ accumulation, potentiated TMZ's tumoricidal effects, and prolonged animal survival. A critical future challenge will be determining the optimal clinical dosage of VLS‐101, particularly in combination with TMZ or other emerging therapies, to maximize drug delivery and antitumor efficacy.

This study has several limitations. First, drug treatment experiments were conducted exclusively in implanted mouse GBM models, which may not fully recapitulate the genetic heterogeneity, microenvironmental complexity, or therapeutic response of human GBM. Second, our analysis of GBM vascular leakage relied on histological assessments at a single time point; advanced in vivo imaging techniques (e.g., dynamic contrast‐enhanced magnetic resonance imaging or intravital microscopy) will be required to characterize the temporal dynamics of vascular function and GDEC behavior following ROR1‐targeted therapy. Third, while we demonstrated that WNT5A‐ROR1 binding activates WNT signaling to promote GDEC formation and suppress endothelial adhesion molecule expression, the exact molecular mechanisms linking WNT signaling to GDEC function (e.g., barrier integrity, proliferation) and adhesion molecule regulation remain incompletely defined. Finally, preclinical findings must be validated in clinical trials to evaluate the safety and efficacy of ROR1‐targeted approaches in GBM patients.

In summary, our study identifies ROR1 as a critical driver of GSC‐to‐GDEC differentiation in hypoxic GBM. We further revealed that TAM‐derived WNT5A is an important ligand for ROR1 in this process, and that serum WNT5A may serve as a clinically useful biomarker for GBM. The ROR1‐targeting ADC VLS‐101 emerges as a promising therapeutic agent, as it induces GBM vascular normalization, enhances TMZ delivery, and improves treatment efficacy. These findings support the further development of ROR1‐targeted strategies in preclinical models and their eventual translation to clinical trials for GBM.

## 4 Experimental Section

### 4.1 Clinical Glioma Samples

Human glioma samples were obtained from Beijing Tiantan Hospital and The First Affiliated Hospital of Fujian Medical University, with approval from the Ethics Committee of the two hospitals and informed consent from patients or their guardians (No. KY2024‐171‐02). The procedures followed the ethical guidelines outlined in the Declaration of Helsinki.

### 4.2 Cell Culture

The patient‐derived glioblastoma stem cell (GSCs) BNI2‐4 (GSC‐1) and BNI22‐1 (GSC‐2) were obtained from the Beijing Neurosurgical Institute as previously described [11, 54]. The GL261‐luciferase positive (GL261‐luc) cells were obtained from Nanjing SaiHongRui Life Sciences Co., Ltd (Jiangsu, China, SHC6031, RRID: CVCL_X986). They were cultured in DMEM/F12 medium (Gibco) supplemented with B27 supplement without vitamin A (211621, NEST Biotechnology), 20 ng/mL human EGF (CM120, purchased from Chamot Biotechnology Co., Ltd.), 20 ng/mL human FGF (CM091, purchased from Chamot Biotechnology Co., Ltd.), MEM nonessential amino acids (1X, Gibco), and antibiotic‐antimycotic (1X, Gibco). GSCs were maintained in a spherical, stem cell‐like growth pattern and dissociated for passaging every 3–5 days using Accutase (StemCell Technologies). GDECs were generated through the differentiation of GSCs. Briefly, GSCs neurospheres were enzymatically dissociated into single cells, which were then cultured in endothelial cell medium (ECM) on Matrigel‐coated (1%, Corning) dishes for 2 weeks. The endothelial cell medium (Sciencell) consists of ECM basal medium supplemented with 5% fetal bovine serum (Cat. No. 0025), 1% Endothelial Cell Growth Supplement (Cat. No. 1052), and 1% penicillin/streptomycin (Cat. No. 0503). The human monocytic leukemia cell line THP‐1 was obtained from the Shanghai Cell Bank of the Chinese Academy of Sciences (Shanghai, China, SCSP‐567, RRID: CVCL_0006). CD14+ monocytes were derived from peripheral blood mononuclear cells (PBMCs) of healthy donors and isolated via positive selection using anti‐CD14 microbeads (Miltenyi Biotec) with magnetic activation. Both THP‐1 cells and CD14^+^monocytes were cultured in RPMI‐1640 medium (Gibco) supplemented with 10% fetal bovine serum (Gibco). All cells were free of mycoplasma contamination for the described experiments in this study, maintained in a humidified cell culture incubator at 37°C with 5% CO_2_, with regular medium changes and passaging performed as needed.

### 4.3 Tube Formation Assay

Matrigel (Corning) was dispensed into each well of a 48‐well Transwell plate (Corning) using a chilled pipette tip. The plate was incubated at 37°C for 30 min to allow matrix polymerization. Following gel solidification, 4 × 10^4^ GSCs resuspended in 250 µL endothelial basal medium (Sciencell) were seeded onto the Matrigel‐coated wells. Plates were then incubated at 37°C in a humidified atmosphere containing 5% CO_2_ for 24 h to facilitate tube formation. At least 3 brightfield images per well were captured using an inverted microscope (Zeiss), and quantification of total branch length was performed using ImageJ software.

### 4.4 Immunocytochemical Staining

Dissociated single‐cell GSCs/GDECs were cultured on poly‐L‐lysine‐coated confocal dishes (NEST Biotechnology) for CD133/CD31, TJP1, β‐catenin, and ROR1/WNT5A staining/costaining. Following experimental treatments, cells were fixed with 4% paraformaldehyde for 15 min and blocked with BSA (Solarbio) for 1 h. Cells were then incubated overnight at 4°C with the following primary antibodies (Table S1). All primary antibodies were used at the concentrations recommended by the manufacturers. After washing with TBST, cells were incubated with Alexa Fluor 594‐ or 488‐conjugated secondary antibodies for 1 h at room temperature. Nuclei were counterstained with DAPI (Solarbio). Finally, samples were mounted onto glass slides using an antifade aqueous mounting medium. Images were acquired using a Zeiss Axio Observer Z1 confocal microscope.

### 4.5 RNA‐seq

For RNA sequencing, each biological replicate consisted of 2 × 10^6^ cells. Total RNA was isolated with the RNeasy Mini Kit (Qiagen) and treated with RNase‐Free DNase (QIagen) to remove genomic DNA. Following poly(A) selection, libraries were constructed by the NEB Ultra II Directional RNA Kit. Sequencing was performed on an Illumina NovaSeq platform.

### 4.6 RNA‐seq Analysis

Raw sequencing reads were subjected to quality control using FASTQ. The clean reads were then mapped to the human reference genome (GRCh38/hg38) with STAR. Then, a count matrix was generated and subsequently normalized and analyzed for differential expression using the DESeq2 package. Differentially expressed genes (DEGs) were defined as those exhibiting an absolute log2 fold change > 1 and an adjusted *p*‐value < 0.05. Genes were ranked in descending order based on their Log2 fold change values to generate an ordered gene list. Enrichment Analysis was subsequently performed using the fgsea and Gene Set Variation Analysis (GSVA) packages on the gene list to assess the activation status of 50 Hallmark gene sets and a custom ROR1+ GDEC signature across comparative groups.

### 4.7 ScRNA‐seq

ScRNA‐seq data from GBM patients were integrated and processed using the Seurat package in R as previous description [20]. Low‐quality cells were filtered out, and then the gene expression matrix was normalized further analysis. Highly variable genes were selected for dimensionality reduction through principal component analysis (PCA). Nonlinear visualization was achieved using Uniform Manifold Approximation and Projection (UMAP). Cell clusters were identified by applying the FindNeighbors and FindClusters functions. Major cell populations were annotated based on canonical marker genes: PTPRC (immune cells); *SOX2* and *PTPRZ1* (malignant cells); *CD14, AIF1, FCER1G, FCGR3A, TYROBP, CSF1R, S100A8*, and *S100A9* (myeloid cells); *CD2, CD3E*, and *CD3G* (T cells); MBP and PLP1 (glias). Tumor cells and myeloid cells were extracted for secondary analysis, and cell subtypes were grouped by expression of target genes (*CD31, CD34, ROR1, WTAP, WNT5A*). To investigate functional characteristics of cell subpopulations, Hallmark pathway activities were assessed using GSVA and visualized was by the ggplot2 package.

### 4.8 Reverse Transcription‐Quantitative PCR (RT‐qPCR)

Total RNA was isolated from cells/tissues using the FastPure Cell/Tissue Total RNA Isolation Kit V2 (Vazyme, RC112‐01, China), followed by cDNA synthesis with NovoScript Plus All‐in‐one cDNA Synthesis SuperMix (Cat No. E047; Novoprotein, Shanghai, China). Quantitative real‐time PCR was conducted with NovoStart SYBR qPCR SuperMix Plus (Cat No. E096; Novoprotein, Shanghai, China). PCR tubes (MT‐060‐S, PT‐PCR‐0208‐A) were provided by Jiangsu Maige Biotechnology Co., Ltd. Gene expression levels were quantified via the 2^−ΔΔCt^ method, with all target genes analyzed in triplicate or more replicates. Primer sequences are provided in Table S2.

### 4.9 Western Blot

Whole‐cell protein lysates were harvested using Total Protein Extraction Maxi Kit (Cat:BC3710, Beijing Solarbio Science & Technology Co., Ltd.). Protein concentrations were determined using a BCA protein assay kit (KGB2101‐500, KeyGEN BioTECH). Protein lysates were separated by SDS‐PAGE (GenScript, M00662 and M00664) and subsequently transferred onto PVDF membranes (Millipore, 0000327117). After incubation with specific primary antibodies (Table S1), the membranes were incubated with HRP‐conjugated secondary antibodies at room temperature for 1 h. Protein bands were visualized using an ECL Western Blotting detection reagent (KGC4601‐200, KeyGEN BioTECH).

### 4.10 Immunohistochemistry and IF Staining

As previously reported [56, 57, 58], Tumor tissues were fixed overnight in 4% paraformaldehyde. Subsequently, samples were dehydrated, embedded in paraffin, and sectioned consecutively at 5 µm thickness using a microtome. Sections were deparaffinized prior to staining. For histological assessment, sections were stained with Hematoxylin and Eosin (H&E) to confirm pathological diagnosis. For immunohistochemical (IHC) analysis, antigen retrieval was performed by boiling tissue sections in Tris‐EDTA buffer (Solarbio). After blocking with goat serum (ZSGB‐BIO), sections were incubated overnight at 4°C with specific primary antibodies. All primary antibodies were diluted to the manufacturer's recommended concentration using antibody diluent (ZSGB‐BIO). Following primary antibody incubation (Table S1), sections were incubated with horseradish peroxidase (HRP)‐conjugated secondary antibodies (ZSGB‐BIO) at room temperature for 1 h. Antigen‐antibody complexes were visualized using 3,3'‐diaminobenzidine (DAB) chromogen (ZSGB‐BIO), and nuclei were counterstained with hematoxylin. Finally, sections were dehydrated and mounted with neutral resin. For immunofluorescence (IF) analysis, sections were incubated with Alexa Fluor 488‐ or 594‐conjugated secondary antibodies (Y1104, Y1007, Beijing LABLEAD Inc.) after primary antibody incubation. Nuclei were counterstained with DAPI (Solarbio). Sections were mounted using an antifade aqueous mounting medium. All images were acquired at 40x magnification using a NanoZoomer digital slide scanner and analyzed using ImageJ software.

### 4.11 Enzyme Linked Immunosorbent Assay

ELISA was performed to measure the concentration of WNT5A in patients' peripheral blood, supernatants from GSCs, TAMs, and coculture medium according to the manufacturer's instructions (R&D Systems).

### 4.12 Proximity Ligation Assay

Duolink PLA was performed using the kit (Sigma, DUO92101‐1KT). Cells were seeded on chambered slides (NEST Biotechnology, 230118), followed by fixation and permeabilization. Subsequently, samples were blocked with Duolink Blocking Solution at 37°C for 60 min in a humidified chamber. Primary antibodies against WNT5A and ROR1, diluted to optimal concentrations in Duolink Antibody Diluent, were applied to the samples and incubated under optimized conditions, ensuring coverslips remained hydrated to minimize background interference. After primary antibody incubation, slides were washed twice with 1× Wash Buffer A at room temperature, 5 min per wash. Diluted Duolink PLUS and MINUS PLA Probes were then added, and samples were incubated at 37°C for 1 h. Following probe incubation, slides were washed as described above. Ligase was added at a 1:40 dilution in 1× Ligation Buffer, and samples were incubated at 37°C for 30 min. After washing, slides were treated with 1× Amplification Buffer and polymerase, followed by incubation at 37°C for 100 min. Samples were washed twice with 1× Wash Buffer B at RT (10 min per wash) and once with 0.01× Wash Buffer B for 1 min. CoraLite488‐conjugated phalloidin (Proteintech, PF00001) was added for 10 min at RT, then washed again. Samples were mounted with DAPI‐containing Duolink mounting medium and, after a 15‐min settling period, imaged using a 40× objective on a fluorescence or confocal microscope.

### 4.13 Gene Manipulation

Scrambled‐siRNA and HuR‐siRNA were purchased from Genepharma (Suzhou, China). siRNA transfections were performed using PolyPlus‐transfection reagent according to the manufacturer's instructions. Lentiviral particles expressing WTAP, ROR1, WTAP‐shRNA, ROR1‐shRNA, scrambled shRNA, and empty vector, AAV_ROR1i (X1.1) and AAV_NC (X1.1) were obtained from GenePharma and GeneChem Co. Ltd. (Shanghai, China). GSCs were transduced with lentiviruses following the supplier's protocol. The sequences of these selected siRNAs and shRNA were summarized in Table S3.

### 4.14 In Vivo Experiment

This study involving animal care and use was approved by the Animal Welfare Ethics Committee of Beijing Neurosurgical Institute (Approval No. 20200002) and was conducted under its supervision throughout the study. BALB/c Nude and C57BL/6J mice (Beijing Vitalstar Biotechnology Co., Ltd., China) were housed in specific pathogen‐free (SPF) barrier facilities at the Animal Experiment Center of Beijing Neurosurgical Institute. Animals were maintained under controlled conditions: 12‐h light/dark cycle, 20°C –24°C ambient temperature, 40%–60% relative humidity, with ad libitum access to food and water. The animal experiments utilized cells transduced with lentiviral vectors carrying overexpression constructs, shRNAs, or control vectors, which were provided by Genepharma or GeneChem Co. Ltd. All orthotopic intracranial tumor models were established using luciferase‐transfected tumor cells. A 5 µL cell suspension was stereotactically injected into the right cerebral cortex. Cell inoculation doses were: 2 × 10^5^ GL261 cells/mouse (GL261 model), 2 × 10^5^ BNI2‐4 cells/mouse (GSC model), 1 × 10^4^ BNI2‐4 cells + 2 × 10^4^ THP‐1 cells/mouse (GSC‐TAM cotransplantation model). All mice underwent weekly bioluminescent imaging (PerkinElmer), body weight monitoring, and clinical assessment. Mice bearing tumors for AAV_ROR1i or AAV_NC treatment were performed 3 intravenous (i.v.) injections (dose range: 5 × 10^11^ to 1 × 10^12^ vg/injection in 100 mL DPBS) at days 4, 7, and 10 after tumor implantation. VLS‐101 (2.5 mg/kg) was administered intravenously (i.v.) once weekly for 3 weeks. And TMZ (5 mg/kg, i.p.) was given for 3 consecutive days. Control groups received PBS with matched volume (i.v. or i.p.).

### 4.15 Vascular Leakage Measurement

Following confirmation of orthotopic tumor establishment by bioluminescent imaging, 3000 MW dextran (Thermo Fisher Scientific, D3306) in sterile saline (15 mg/kg, 150 µL) was administered via tail vein to 3 mice per group. After 1‐h circulation, mice were euthanized, and tumor‐bearing brains were harvested. Tissues were fixed in 4% PFA for 24 h, dehydrated in 15%/30% sucrose gradients, embedded in OCT, and cryosectioned at 10 µm. Sections were rehydrated, washed in PBS, and costained with anti‐CD31 antibody (1:500, R&D Systems), followed by Alexa Fluor 594‐conjugated secondary antibody and DAPI. Samples were mounted with antifade medium. Images were acquired using a Zeiss LSM900 confocal microscope to analyze vascular permeability.

### 4.16 Protein–Protein Interaction Prediction

Protein–protein interaction networks involving ROR1 were predicted using the STRING database (https://string‐db.org/), which contains known and predicted interactions from experimental results, coexpression, and genomic context analysis. AlphaFold3, an advanced deep learning‐based protein structure prediction system, was used to predict and quantify the interaction between ROR1 and WNT5A. The protein sequences of the two human protein from UniProt (ROR1,UniProt: Q01973; WNT5A, UniProt: P41221) were inputted into the model without further modification and performed prediction under default parameters.

### 4.17 Coimmunoprecipitation

For coimmunoprecipitation (Co‐IP) assays, GSCs were counted, and 2 × 10^7^ cells were resuspended in ice‐cold PBS. After three washes with PBS, cells were lysed in 1 mL nondenaturing lysis buffer with protease inhibitors on ice for 30 min. The lysates were centrifuged at 12 000 rpm/min for 30 min at 4°C, and the supernatants were collected. For immunoprecipitation, 50 µL of the lysate was set aside as input control. The experimental group was prepared by incubating 950 µL lysate with 40 µL Protein A/G agarose beads and 5 µg anti‐ROR1 antibody (Table S1), while the IgG control group consisted of 950 µL lysate, 40 µL Protein A/G agarose beads, and 5 µg IgG. All complexes were rotated overnight at 4°C. The beads were then pelleted by centrifugation at 1000×g for 5 min at 4°C, washed three times with lysis buffer, and resuspended in 40 µL 2×SDS loading buffer. Samples were denatured at 100°C for 10 min before being subjected to SDS‐PAGE and immunoblotting analysis.

### 4.18 Spatial Transcriptomic‐seq and Analysis

Spatial transcriptomic (ST) profiling was performed on fresh IDH‐wildtype glioblastoma (GBM) tissues from one patient in The first affiliated hospital of Fujian medical university using the 10x Genomics Visium platform according to the manufacturer's protocol. Briefly, surgical specimens were snap‐frozen in optimal cutting temperature (O.C.T.) compound and sectioned at a thickness of 10 µm onto Visium Spatial Gene Expression slides. Sections were stained with hematoxylin and eosin (H&E) for histological assessment. Samples exhibiting preserved tissue morphology and high RNA quality (RNA integrity number ≥ 7) were selected for subsequent processing. Tissue permeabilization was carried out to release RNA, which was then captured by spatially barcoded oligonucleotides. Following mRNA capture, cDNA synthesis and amplification were performed to construct sequencing libraries. Final libraries were sequenced on an Illumina platform under a paired‐end 150 bp reading strategy (Dynamic Biosystems, Suzhou). Raw spatial sequencing data were processed using Space Ranger (10x Genomics) for quality control. This workflow included tissue image alignment, barcode/UMI counting, and alignment to the reference genome (GRCh38). A gene expression matrix incorporating spatial coordinates for each sequencing spot was generated. Subsequent analyses were performed using Seurat (v.4.3.0). Data were normalized and scaled to mitigate technical noise, followed by dimensionality reduction through PCA. Clustering was performed via the Louvain algorithm for community detection, with results visualized in two dimensions using UMAP. The spatial expression distribution of ROR1+GDEC (*PTPRZ1, PROM1, CD44, NES, ROR1, PECAM1*) and WNT5A+TAM (WNT5A, CD14, C1QA, CD68, CD86) populations from scRNA‐seq was analyzed using the AddModuleScore function.

### 4.19 MeRIP‐seq

Total RNA was extracted using TRIzol Reagent and quantified with a NanoDrop 2000 spectrophotometer. For each biological replicate, 200 µg of total RNA was subjected to ribosomal RNA depletion using the RiboMinus Eukaryote Kit v2 (A15020, Invitrogen) according to the manufacturer's protocol. The ribodepleted RNA was fragmented into ∼100 nucleotide fragments using RNA Fragmentation Reagents (AM8740, Invitrogen). Aliquots of the fragmented RNA were stored at −80°C as input controls. For immunoprecipitation, the fragmented RNA was incubated with 5 µg of anti‐m^6^A antibody (ab151230, Abcam) in IP buffer for 2 h at 4°C with rotation. The antibody‐RNA complexes were then captured by incubation with prewashed Pierce Protein A/G Magnetic Beads (88 803, Thermo Scientific) overnight at 4°C. The purified RNA was recovered by ethanol precipitation for subsequent sequencing.

### 4.20 RIP‐qPCR

GSCs were lysed using IP Lysis Buffer provided in the Magna RIP Kit (17–700, Millipore). The lysates were aliquoted for three groups: experimental group, IgG control group, and 10% of the total lysate was reserved as input control. Magnetic beads were precoated with 5 µg of anti‐WTAP antibody (abcam, ab195380), anti‐HuR antibody (abcam, ab200342), or IgG for 1 h at 25°C. For immunoprecipitation, the antibody‐bead complexes were incubated with the corresponding lysates overnight at 4°C with rotation. After immunoprecipitation, the beads were washed six times with RIP Wash Buffer, followed by treatment with 50 µg/mL Proteinase K (P8107S, NEB) at 55°C for 30 min. The coprecipitated RNA was purified using phenol‐chloroform extraction and ethanol precipitation. The purified RNA was subjected to reverse transcription, and the enrichment of target RNAs was quantified by qPCR.

### 4.21 RNA Stability Assays

GSCs were plated in 6‐well plates at a density of 5 × 10^5^ cells per well and incubated in a humidified cell culture incubator for 24 h. After stabilization, actinomycin D (5 µg/mL, Cell Signaling Technology) was added to the culture medium to block new RNA transcription. Cells were collected at 0, 2, 4, and 6 h following actinomycin D treatment, with all cultures maintained at 37°C in a 5% CO_2_ atmosphere throughout the experimental period. Total RNA was isolated using TRIzol reagent according to the manufacturer's instructions. qPCR was performed to determine the relative abundance of ROR1 mRNA, with bacterial 16S rRNA used as an internal control for normalization. The half‐life of ROR1 mRNA was calculated by fitting the degradation curve to a linear regression model using GraphPad Prism software.

### 4.22 HPLC

The cerebral hemisphere containing tumor and adjacent tissue, and other organs (heart, kidney, liver) were harvested and homogenized following established protocols [42], and subsequently analyzed temozolomide concentration using HPLC. Specifically, 400 mg of tissue was homogenized with 200 µL of 10 mM ammonium acetate buffer (pH 3.5), 200 µL of 100 mM zinc sulfate solution, and 400 µL of methanol. The homogenate was centrifuged at 2862 g for 10 min at room temperature, and the supernatant was transferred to an Eppendorf tube for further centrifugation at 21 885 g for 15 min. The secondary supernatant was collected, lyophilized, and reconstituted in 100 µL of 15% methanol‐0.2% acetic acid aqueous solution. A 50 µL aliquot of the reconstituted sample was injected into the HPLC system for analysis.

### 4.23 Transmission Electron Microscopy (TEM)

TEM was performed to analyze the vascular structure in GBM xenograft tumor tissues. Mice were anesthetized and transcardially perfused sequentially with PBS followed by TEM fixation buffer (4% paraformaldehyde and 2.5% glutaraldehyde in 0.1 M cacodylate buffer). Harvested brain tumor tissues were postfixed in the same TEM buffer overnight at 4°C. After fixation, samples were embedded in Eponate 12 resin supplemented with DMP‐30 (Ted Pella) according to standard protocols. Ultrathin sections (80 nm thickness) were cut and stained with uranyl acetate and lead citrate for contrast enhancement. Images were acquired using an transmission electron microscope (HITACHI HT7700).

### 4.24 Glioblastoma Organoids (GBOs) Generation

GBOs were generated following an established protocol [59]. Briefly, freshly resected glioblastoma specimens were placed in a sterile glass dish containing H+GPSA medium, composed of Hibernate A (Thermo Fisher Scientific, A1247501), 1× GlutaMax (Thermo Fisher Scientific, 35050061), and 1× Antibiotic‐Antimycotic (Thermo Fisher Scientific, 15240062), for initial processing. The tissues were minced into pieces approximately 0.5–1 mm in diameter using a sterile disposable scalpel and rinsed with H+GPSA medium to eliminate cellular debris. Subsequently, the pieces were incubated with 1× RBC lysis buffer (Thermo Fisher Scientific, 00433357) under gentle rotation for 10 min to remove contaminating erythrocytes at room temperature. After aspiration of the lysis buffer, the pieces were washed again with H+GPSA medium. Then, tumor tissue pieces were plated in ultralow attachment 6‐well plates (Corning) with 4 mL/well of GBO culture medium. The medium consisted of a 1:1 mixture of DMEM/F12 (Thermo Fisher Scientific, 11330032) and Neurobasal Medium (Thermo Fisher Scientific, 21103049), supplemented with 1× GlutaMax (Thermo Fisher Scientific, 35050061), 1× NEAAs; (Thermo Fisher Scientific, 11140050), 1× Penicillin‐Streptomycin (Sigma, V900929), 1× N2 Supplement (Thermo Fisher Scientific, 17502048), 1× B27 Supplement minus vitamin A (211621, NEST Biotechnology), 1× 2‐mercaptoethanol (Sigma, M3148), and 2.5 mg/mL human insulin (SantaCruz, SC‐360248). Cultures were maintained under continuous orbital shaking (75 rpm) in a sterile incubator at 37°C with 5% CO_2_ and 90% humidity.

### 4.25 Statistical Analysis

Statistical analyses were performed using GraphPad Prism (v8.0) and R (v4.2.1). Data normality was assessed with the Kolmogorov–Smirnov test. For comparisons, normally distributed data were analyzed by two‐tailed unpaired *t*‐test or analysis of variance (ANOVA), while non‐normal data were analyzed by two‐tailed Wilcoxon test. Sample size (n) for each statistical analysis was at least three. Survival was analyzed by Cox regression and Kaplan–Meier curves with log‐rank test. Data from at least three independent biological replicates are presented as mean ± standard deviation (SD) or mean ± standard error of the mean (SEM), as specified. Statistical significance was defined as follows: *p < 0.05, **p < 0.01, ***p < 0.001, and ****p < 0.0001.

## Author Contributions

R.C., T.J., and D.K. contributed to conceptualization. X.C., B.P., Y.L., J.W., L.Z., Y.C., Y.S., and H.S. contributed to methodology. J.W., L.Z., Y.C., Y.S., H.S., H.C., J.C., Z.W., Q.C., and Y.W. performed the investigation. X.C. and J.W. contributed to visualization. R.C., T.J., and D.K. contributed to supervision. R.C., X.C., and B.P. contributed to the writing of the original draft. All the authors contributed to writing, reviewing, and editing.

## Funding

The Noncommunicable Chronic Diseases‐National Science and Technology Major Project (No. 2024ZD0525300); the National Natural Science Foundation of China (Nos. 82272867 and 82403757); High‐level Talents Research Start‐up Project of the First Affiliated Hospital, Fujian Medical University (No. YJRC4194); NSFC/RGC Collaborative Research Scheme (No. 82261160578); Beijing Municipal Health Commission (No. JYY2023‐2); Beijing Key Laboratory of Drug Innovation for Neuro‐Oncology (No. Z241100009024044); Fundings from Beijing Neurosurgical Institute (No. 11000025T000003319495); Fundings from Beijing Tiantan Hospital for Supporting Young talent Researchers; China Postdoctoral Science Foundation (Nos. 2025T180557 and 2024M762188); Beijing Postdoctoral Research Funding.

## Conflicts of Interest

The authors declare no conflicts of interest.

## Supporting information




**Supporting File 1**: advs73773‐sup‐0001‐SuppMat.docx.

**Supporting File 2**: advs73773‐sup‐0002‐Data.zip.

## Data Availability

All data needed to evaluate the conclusions in the paper are present in the paper and/or the Supplementary Materials. The raw sequence data of scRNA‐seq reported in this paper have been deposited in the Genome Sequence Archive of the BIG Data Center, Beijing Institute of Genomics (BIG), Chinese Academy of Sciences, under accession number HRA002610. Additional data related to this paper may be requested from the authors.
