# Supplementary material for: Targeting WTAP/ROR1/WNT5A‐Mediated Crosstalk Between Glioma Stem Cells and Macrophages to Normalize Tumor Vasculature and Enhance Chemotherapy
Source: Adv Sci (Weinh). 2026 Jan 21;13(21):e20661. doi: 10.1002/advs.202520661 (PMC13073320; doi:10.1002/advs.202520661)
Supplement: Supplementary file 1 — Supporting File 1: advs73773‐sup‐0001‐SuppMat.docx. [file ADVS-13-e20661-s002.docx]

**Supplementary Information**

**Targeting WTAP/ROR1/WNT5A-mediated Crosstalk between Glioma Stem Cells and Macrophages to Normalize Tumor Vasculature and Enhance Chemotherapy**

*Xiaoyong Chen, Bo Pang, Yun Liu, Jiangwei Wang, Lanhui Zheng, Yuzhou Chang, Yingxuan Sun, Haoyuan Sun, Huiyuan Chen, Jiawei Cai, Zanyi Wu, Qing Chang, Yongzhi Wang, Dezhi Kang^*^, Tao Jiang^*^, Ruichao Chai^*^*

X. Chen, B. Pang, Y. Liu, J. Wang, L. Zheng, Y. Chang, Y. Wang, T. Jiang

Department of Neurosurgery, Beijing Tiantan Hospital, Capital Medical University

Beijing 100070, China

E-mail: taojiang1964@163.com

X. Chen, J. Cai, Z. Wu, D. Kang

Department of Neurosurgery, Neurosurgery Research Institute, The First Affiliated Hospital, Fujian Medical University

Fuzhou 350005, China

E-mail: kangdezhi@fjmu.edu.cn

X. Chen, B. Pang, J. Wang, L. Zheng, Y. Sun, H. Sun, H. Chen, Q. Chang, Y. Wang, T. Jiang, R. Chai

Beijing Neurosurgical Institute, Capital Medical University

Beijing 100070, China

E-mail: chairuichao_glia@163.com

J. Cai, Z. Wu, D. Kang

Department of Neurosurgery, National Regional Medical Center, Binhai Campus of the First Affiliated Hospital, Fujian Medical University

Fuzhou 350212, China

L. Zheng

College of Basic Medical Sciences, Dalian Medical University

Dalian 116044‌, China

T. Jiang, R. Chai

Beijing Key Laboratory of Drug Innovation for Neuro-Oncology

Beijing 100070, China

T. Jiang, R. Chai

Beijing Engineering Research Center of Targeted Drugs and Cell Therapy for CNS Tumors

Beijing 102600, China

T. Jiang, R. Chai

Chinese Glioma Genome Atlas network (CGGA) and Asian Glioma Genome Atlas network (AGGA)

Beijing 100070, China


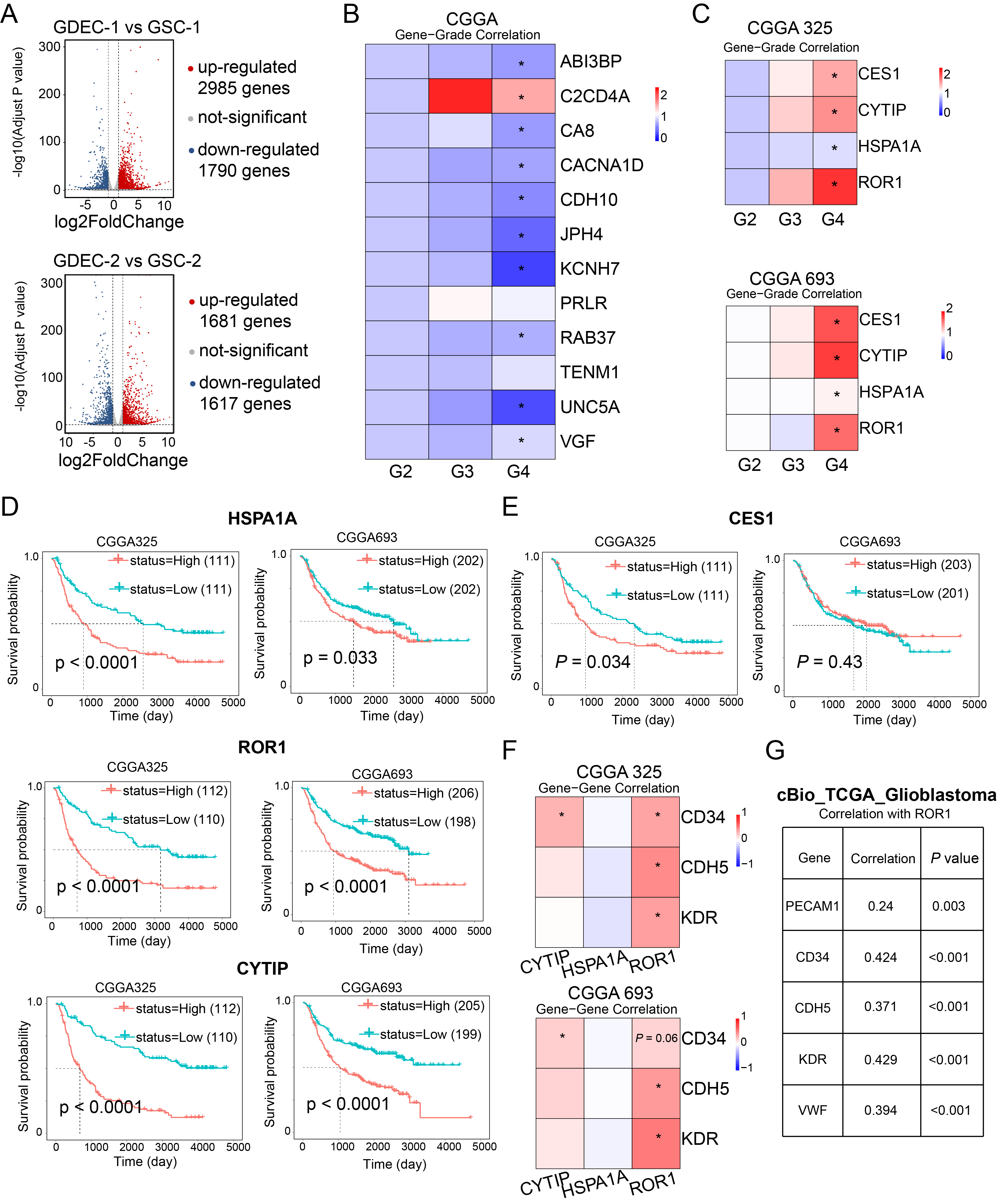
Fig. S1.

**Identification of GDEC differentiation related genes through correlation analysis with glioma grade, patient prognosis, and endothelial markers.** (**A**) Volcano plot of differentially expressed genes (|log2 Foldchange| >1, adjust *P* <0.05) between GDEC and GSC. (**B**) Thirteen candidate genes showing no positive correlation with glioma grade in the CGGA database (MUC5AC showed no result). (**C**) Four candidate genes showing positive correlation with glioma grade in the CGGA 325 and 693 cohort. (**D-E**) Correlation between expression of four candidate genes and prognosis of primary glioma patients in the CGGA 325 and 693 cohort. (**F**) Correlation analysis between three candidate genes and endothelial gene signatures in CGGA 325 and 693 cohort. (**G**) Correlation analysis between ROR1 and endothelial gene signatures in the cBio portal (GBM TCGA). **P* < 0.05, ***P* < 0.01, ****P* < 0.001, and *****P* < 0.0001 for all figures.


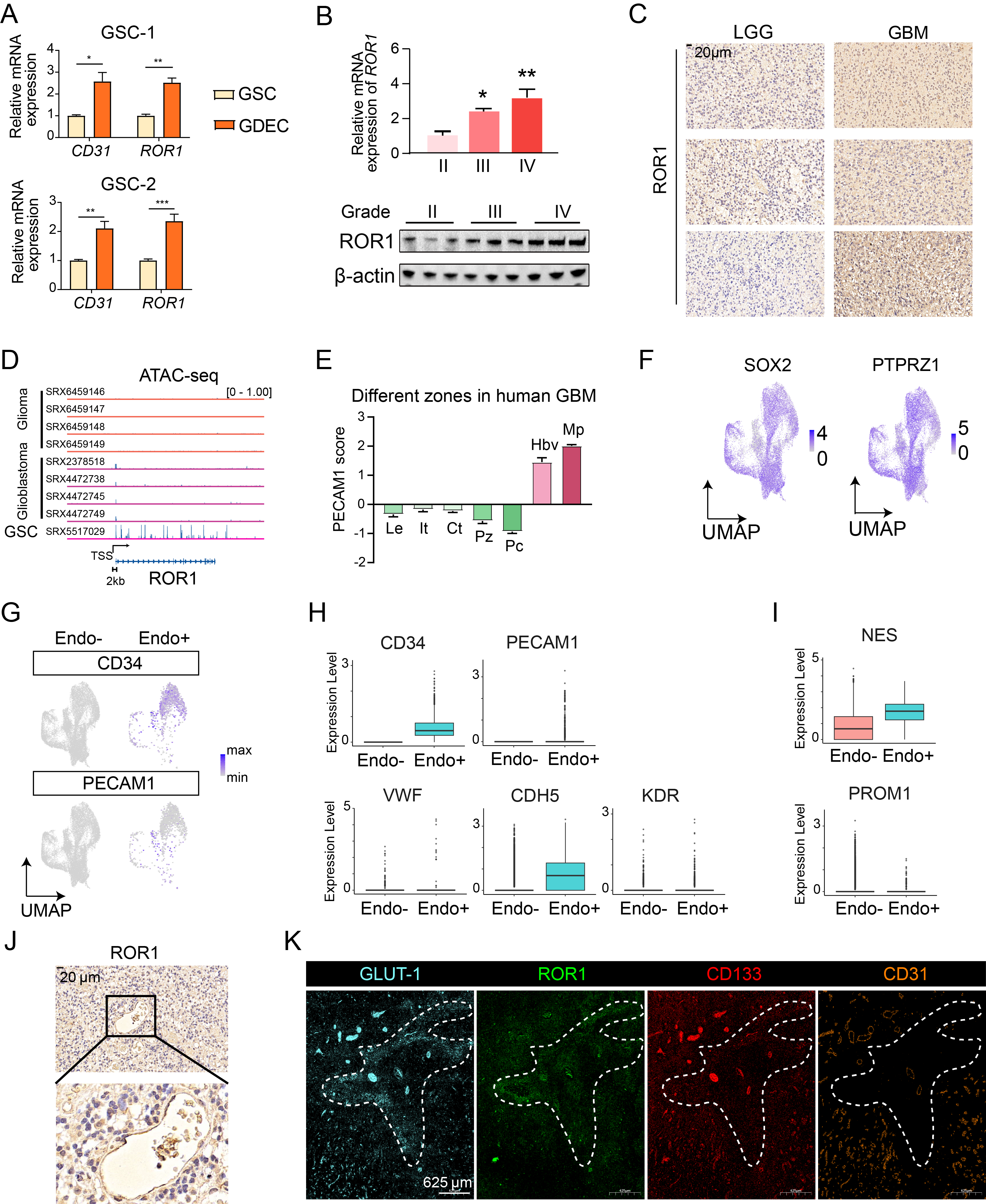


Fig. S2.

**The role of ROR1 in GDEC differentiation and GBM progression.** (**A**) CD31 and ROR1 mRNA expression in GSC and GDEC. (**B**) ROR1 mRNA (top) and protein (bottom) expression in glioma tissues with different histological grades. (**C**) Representative immunohistochemistry images of ROR1 protein in gliomas with different histological grades. (**D**) ATAC-seq signals for ROR1 in low-grade glioma, GBM, and GSCs. (**E**) Bar chart of expression level of PECAM1 in Ivy GAP spatial RNA-seq from GBM patients. (**F**) Feature plot of SOX2 and PTPRZ1 in tumor cells from scRNA-seq of 6 GBM patients. (**G**) Feature plots showing expression of *CD31*, and *CD34* in Endo− and Endo+ GBM cells. (**H**) Box plots showing expression of stemness genes in Endo− and Endo+ GBM cells. (**I**) Box plots showing expression of stemness genes in Endo− and Endo+ GBM cells. (**J**) Representative immunohistochemistry images of ROR1 protein in GBM. (**K**) Multiplex immunohistochemistry of tissue section from GBM patient for GLUT-1, ROR1, CD133, CD31, and DAPI. The hypoxic necrotic zone is demarcated by white dotted lines. **P* < 0.05, ***P* < 0.01, ****P* < 0.001, and *****P* < 0.0001 for all figures.


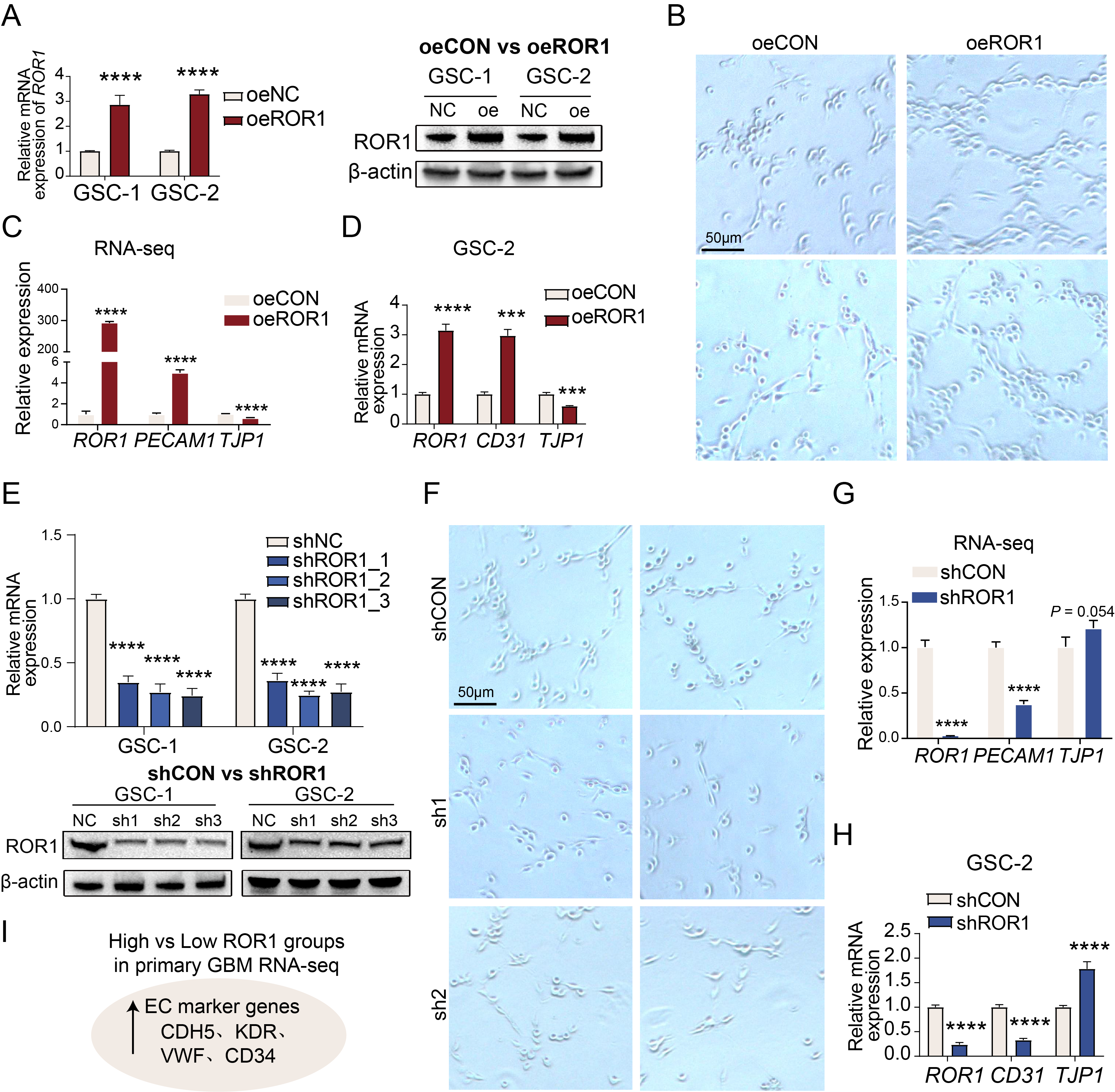


Fig. S3.

**ROR1 modulate GDEC differentiation in vitro.** (**A**) ROR1 mRNA (left) and protein (right) expression in oeROR1 and oeCON GSCs. (**B**) The images of oeROR1 and oeCON GSCs in tube formation assay. (**C**) RNA-seq analysis of ROR1-overexpressing GSCs (oeROR1) versus controls (oeCON) shows significantly elevated *ROR1* and *PECAM1* expression, with significantly reduced *TJP1* levels. (**D**) ROR1, CD31, and TJP1 mRNA expression in oeROR1 and oeCON GSC-2. (**E**) ROR1 mRNA (top) and protein (bottom) expression in shCON, ROR1_sh1, ROR1_sh2, and ROR1_sh3 GSCs. (**F**) The images of shCON, ROR1_sh1, and ROR1_sh2 GSCs in tube formation assay. (**G**) RNA-seq analysis of ROR1-knockdown GSCs (shROR1) versus controls (shCON) shows significantly reduced *ROR1* and *PECAM1* expression, with elevated *TJP1* levels. (**H**) ROR1, CD31, and TJP1 mRNA expression in shROR1 and shCON GSC-2. (**I**) Differential gene analysis (|log2 Fold change| > 1, adjust *P* <0.05) between ROR1-high and ROR1-low groups in primary GBM patients from CGGA cohorts. Bar chart and data are presented as the mean ± SD or mean ± SEM and were analyzed with Student’s t test or one-way ANOVA.**P* < 0.05, ***P* < 0.01, ****P* < 0.001, and *****P* < 0.0001 for all figures.


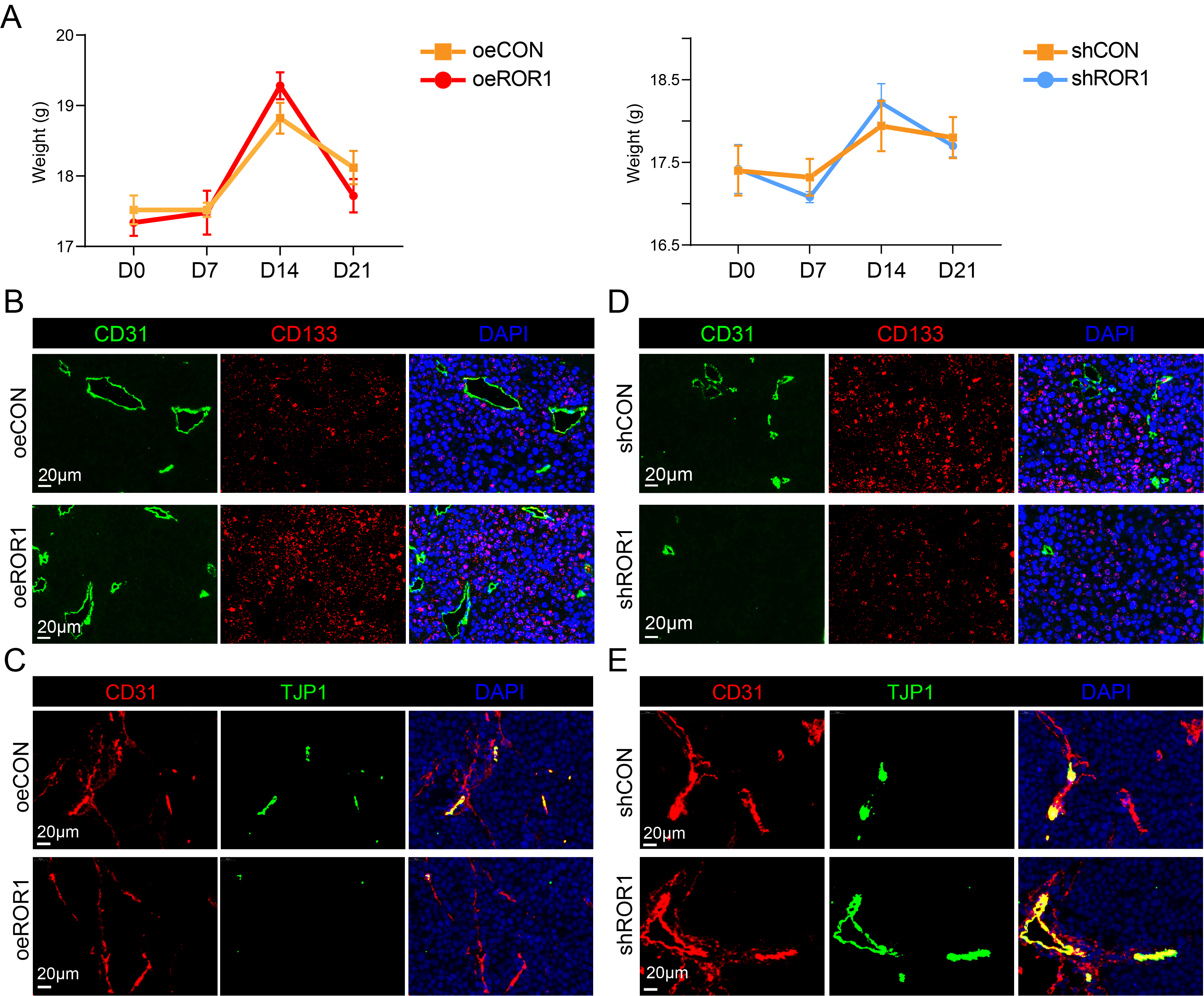


Fig. S4.

**ROR1 modulate GDEC differentiation in vivo.** (**A**) Weight of tumor-bearing mice implanted with oeCON GSCs, oeROR1 GSCs, shCON GSCs, shROR1 GSCs, separately. (**B**) Immunofluorescence staining of CD31, CD133, and DAPI in GBM tissue from tumor-bearing mice implanted with oeCON GSCs or oeROR1 GSCs. (**C**) Immunofluorescence staining of CD31, TJP1, and DAPI in GBM tissue from tumor-bearing mice implanted with oeCON GSCs or oeROR1 GSCs. (**D**) Immunofluorescence staining of CD31, CD133, and DAPI in GBM tissue from tumor-bearing mice implanted with shCON GSCs or shROR1 GSCs. (**E**) Immunofluorescence staining of CD31, TJP1, and DAPI in GBM tissue from tumor-bearing mice implanted with shCON GSCs or shROR1 GSCs.


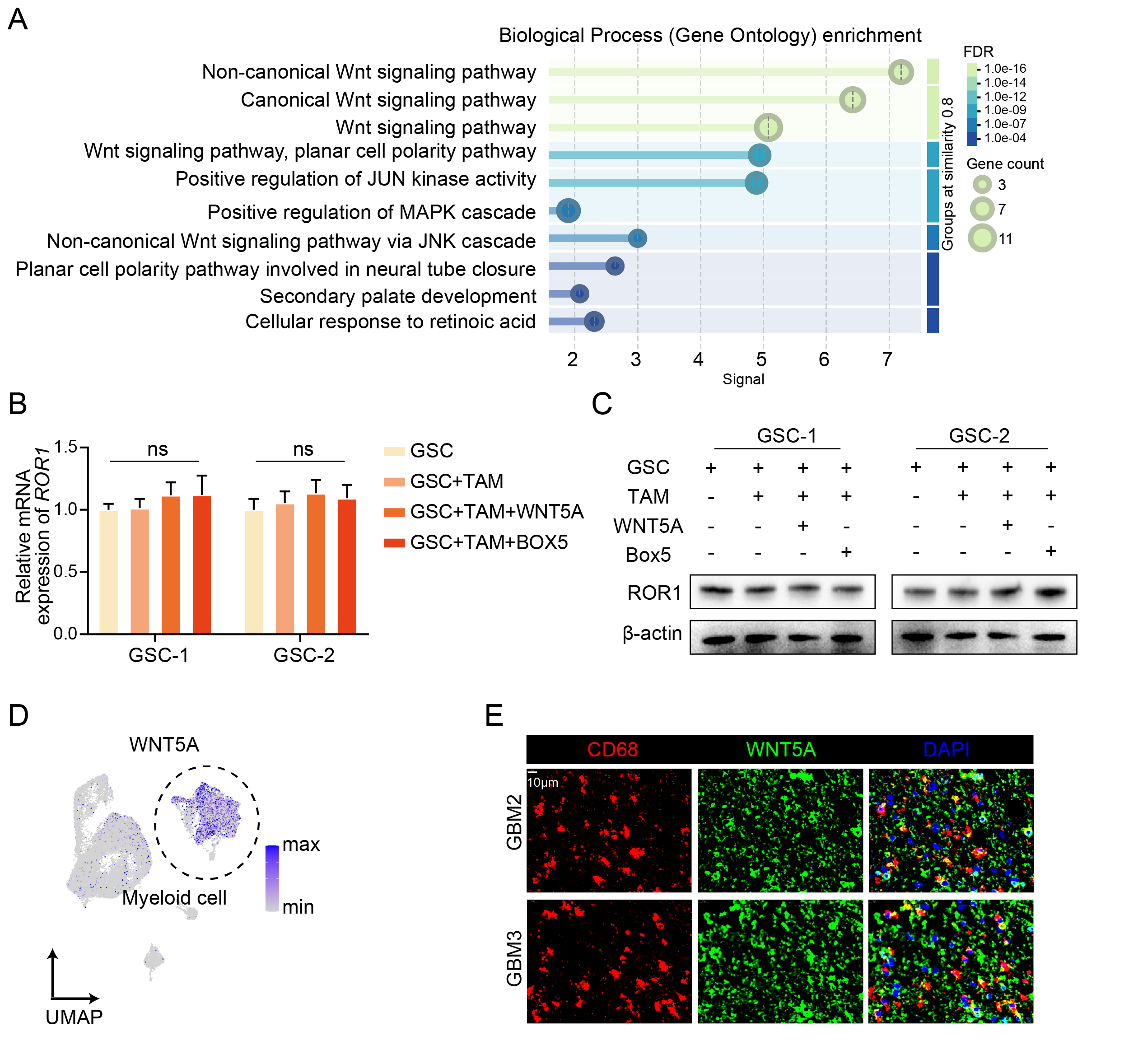


Fig. S5.

**Biological process enrichment based on ROR1 and the primary source of WNT5A.** (**A**) Biological process enrichment based on ROR1. (**B**) WNT5A mRNA expression in GSC with PBS, co-culture of macrophages, co-culture of macrophages and exogenous WNT5A, co-culture of macrophages and WNT5A antagonist (Box5), respectively (n=4). (**C**) WNT5A protein expression in GSC with PBS, co-culture of macrophages, co-culture of macrophages and exogenous WNT5A, co-culture of macrophages and WNT5A antagonist (Box5), respectively. (**D**) Feature plot of scRNA-seq from 6 GBM patients shows that *WNT5A* is primarily distributed myeloid cells. (**E**) Immunofluorescence staining of CD68, WNT5A, and DAPI in GBM tissue from patients. Bar chart and line chart data are presented as the mean ± SD or mean ± SEM and were analyzed with Student’s t test or one-way ANOVA.


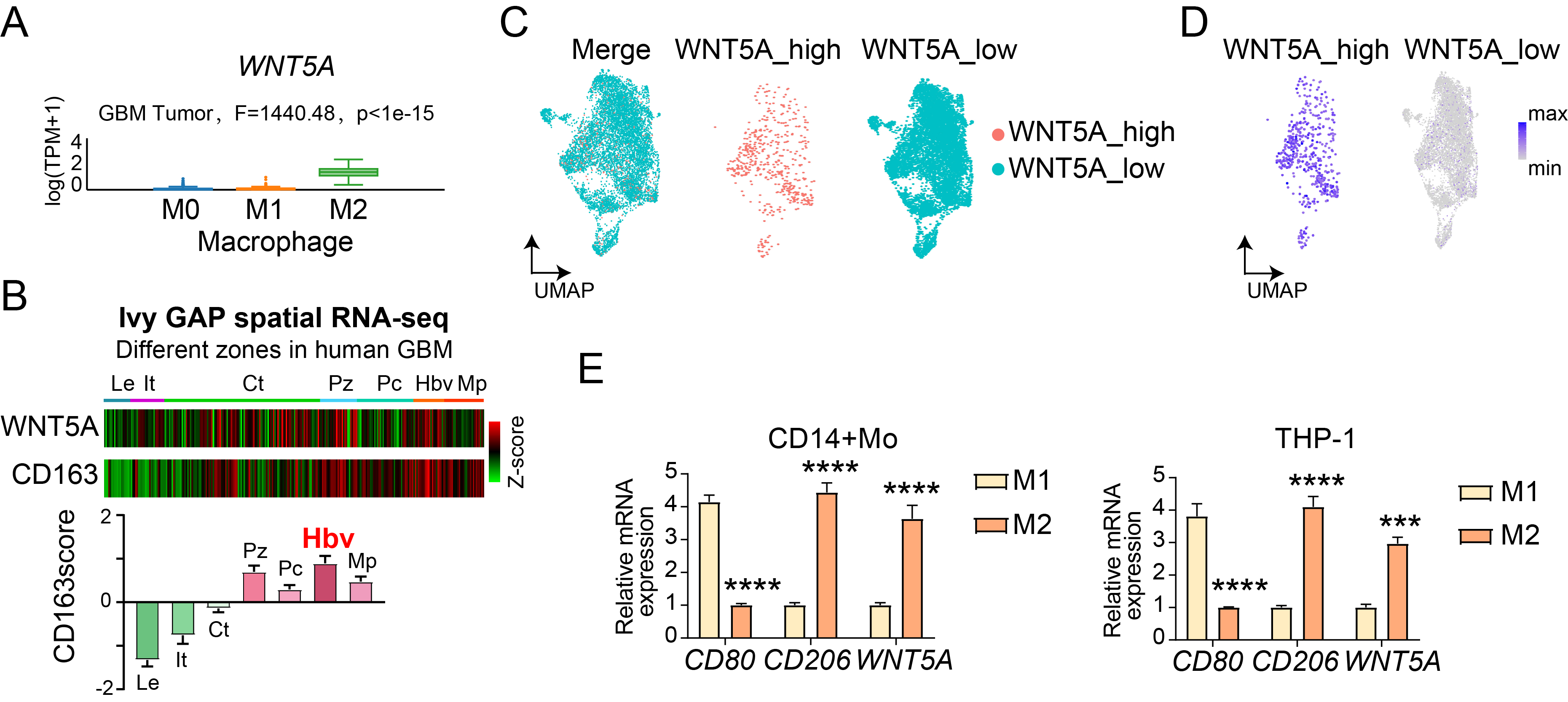


Fig. S6.

**WNT5A is predominantly secreted by M2-like TAMs.** (**A**) Comparison of WNT5A expression in macrophage M0, M1-phenotype, and M2-phenotye cells of GBM tissues from TCGA. (**B**) Heatmap (top) and bar chart (bottom) of expression level of WNT5A and CD163 in Ivy GAP spatial RNA-seq from GBM patients. (**C**) UMAP plot of myeloid cells after dimensionality reduction and grouped by high or low WNT5A expression. (**D**) Feature plot of WNT5A expression in WNT5A_high and WNT5A_low TAMs. (**E**) WNT5A, CD80, and CD206 mRNA expression in M1-phenotype and M2-phenotye TAMs derived from THP-1 and CD14+monocyte. Bar chart data are presented as the mean ± SD or mean ± SEM and were analyzed with Student’s t test. **P* < 0.05, ***P* < 0.01, ****P* < 0.001, and *****P* < 0.0001 for all figures.


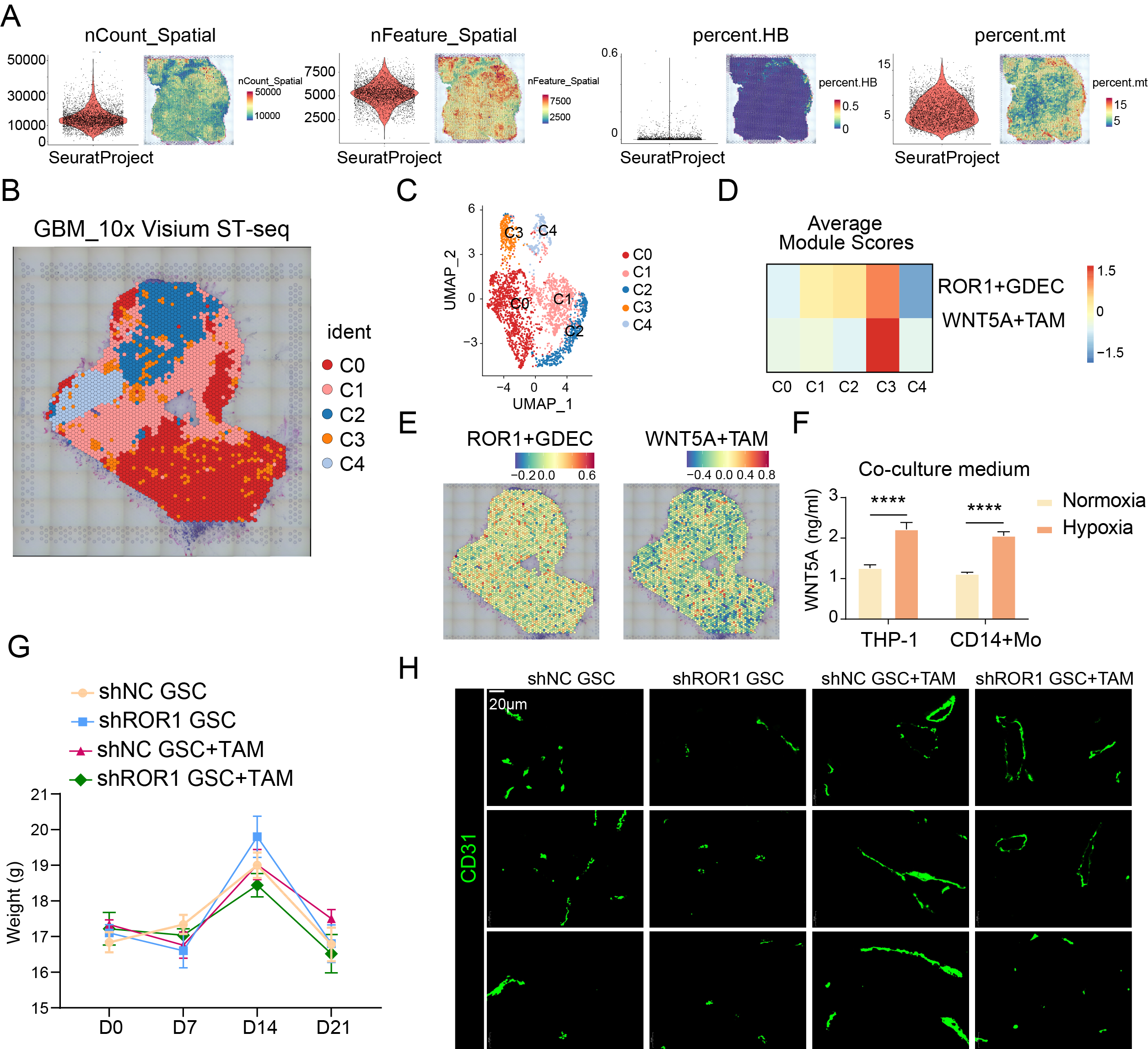


Fig. S7.

**TAMs secrete WNT5A promote GDEC differentiation in vivo. (A)** Quality control of ST-seq of GBM tissue from 1 patient. (**B**) ST-seq data of 1 GBM patient from GSE194329. The sequencing spot were clustered into 5 clusters (C0-C4) after dimensionality reduction. (**C**) UMAP plot of 5 clusters. (**D**) The average module scores of expression level of marker genes of ROR1+GDEC and WNT5A+TAM in 5 spatial clusters. (**E**) Heat map of spatial expression distribution of marker genes of ROR1+GDEC and WNT5A+TAM. (**F**) The WNT5A protein levels in the GSC-macrophage (THP-1 or CD14+monocyte) co-culture medium under normoxic and hypoxic conditions measured by Enzymelinked immunosorbent assay (n=5). **P* < 0.05, ***P* < 0.01, ****P* < 0.001, and *****P* < 0.0001. (**G**) Weight of tumor-bearing mice from 4 groups. (**H**) Immunofluorescence staining of CD31 in GBM tissue from tumor-bearing mice in 4 groups. Bar chart data are presented as the mean ± SD and were analyzed with Student’s t test. **P* < 0.05, ***P* < 0.01, ****P* < 0.001, and *****P* < 0.0001 for all figures.


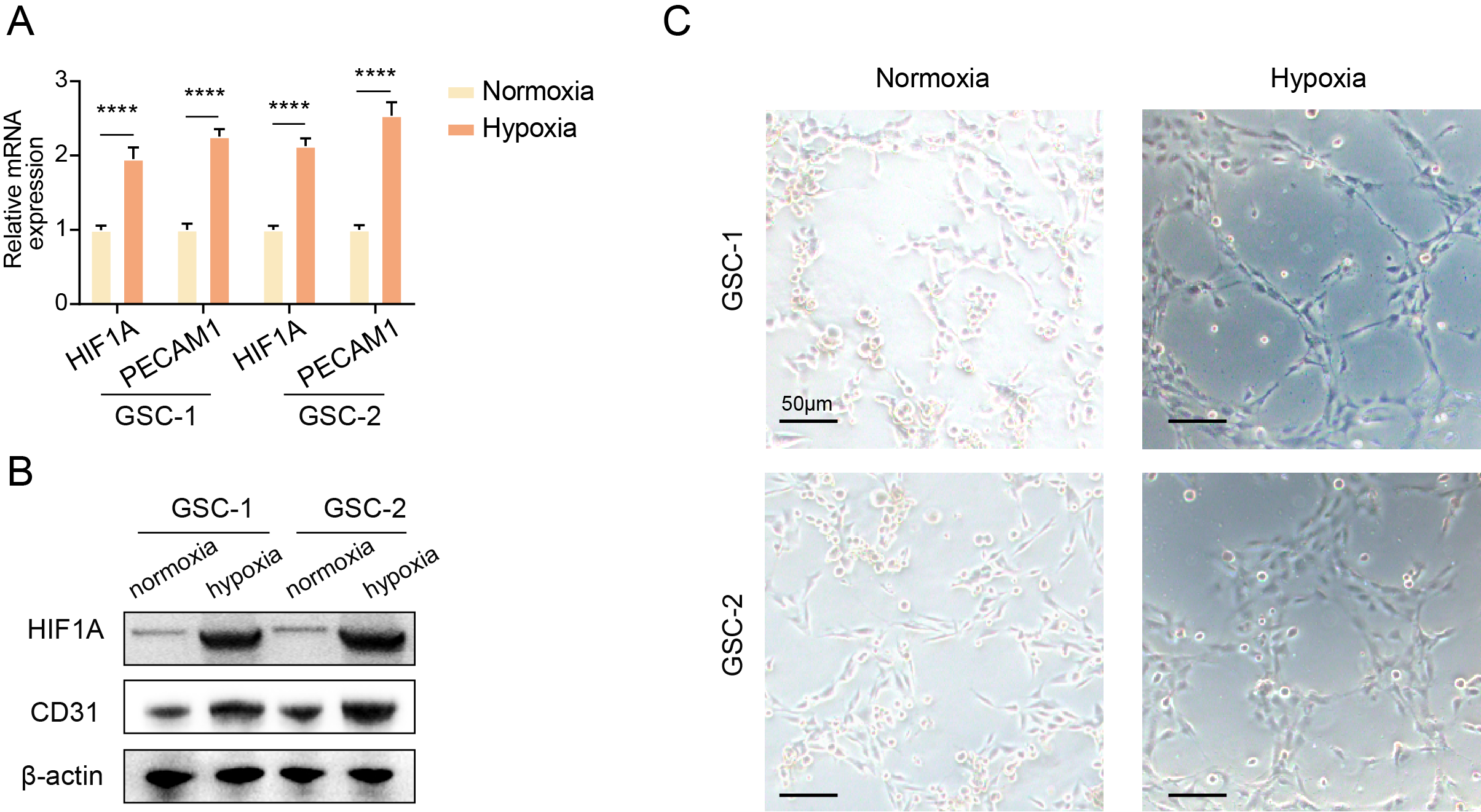


Fig. S8.

**Hypoxia promote GDEC differentiation and abnormal angiogenesis in a co-culture system.** (A) HIF1A and PECAM1 mRNA expression in GSCs under normoxic and hypoxic co-culture conditions (n=4). **P* < 0.05, ***P* < 0.01, ****P* < 0.001, and *****P* < 0.0001. (B) HIF1A and CD31 protein expression in GSC under normoxic and hypoxic co-culture conditions. (C) The images of tube formation assay in GSCs under normoxic and hypoxic co-culture conditions. Bar chart data are presented as the mean ± SD and were analyzed with Student’s t test. **P* < 0.05, ***P* < 0.01, ****P* < 0.001, and *****P* < 0.0001 for all figures.


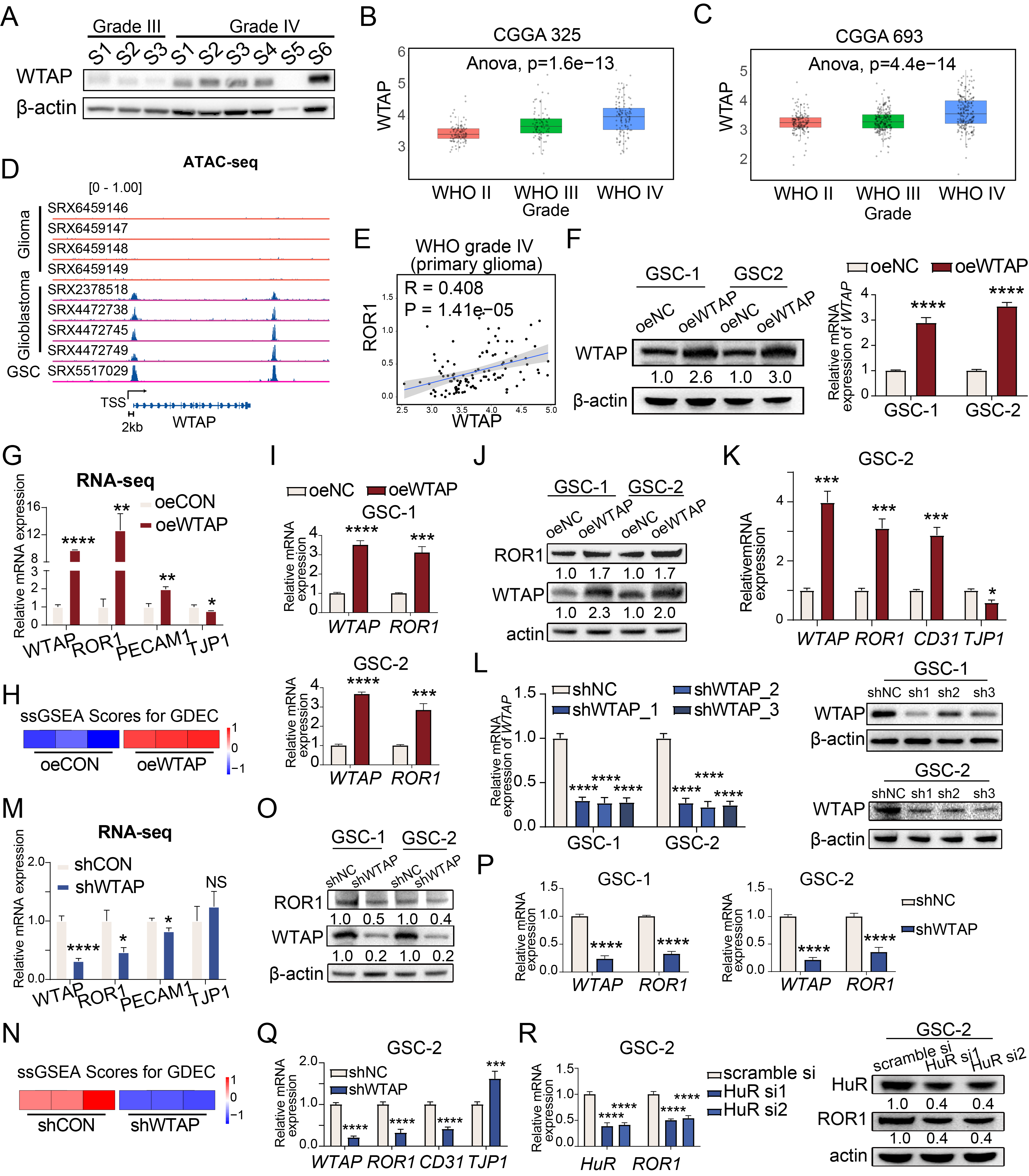


Fig. S9.

**WTAP increases ROR1 stability in GSCs to promote GDEC differentiation through m6A modification in an HuR-dependent manner.** (**A**) WTAP protein expression in grade III and grade IV glioma tissues. WTAP expression of glioma across different WHO grades in the CGGA 325 (**B**) and 693 (**C**) cohort. (**D**) ATAC-seq signals for WTAP in low-grade glioma, GBM, and GSCs. (**E**) Correlation between WTAP and ROR1 in primary GBM from CGGA database. (**F**) WTAP protein (left) and mRNA (right) expression in oeWTAP and oeNC GSCs. (**G**) RNA-seq analysis of WTAP-overexpressing GSCs (oeWTAP) versus controls (oeCON) shows significantly elevated *WTAP*, *ROR1* and *PECAM1* expression, with significantly reduced *TJP1* levels. (**H**) Heatmap of ssGSEA scores for ROR1+GDEC in differential gene analysis between oeWTAP and oeCON GSCs. WTAP and ROR1 mRNA (**I**) and protein (**J**) expression in oeWTAP and oeNC GSCs. (**K**) WTAP, ROR1, CD31, TJP1 mRNA expression in oeWTAP and oeNC GSC-2. (**L**) WTAP mRNA (left) and protein (right) expression in shNC, shWTAP_1, shWTAP_2, and shWTAP_3 GSCs. (**M**) RNA-seq analysis of WTAP-knockdown GSCs (shWTAP) versus controls (shCON) shows significantly reduced *WTAP*, *ROR1* and *PECAM1* expression, with slightly elevated *TJP1* levels. (**N**) Heatmap of ssGSEA scores for ROR1+GDEC (bottom) in differential gene analysis between shWTAP and shCON GSCs. WTAP and ROR1 protein (**O**) and mRNA (**P**) expression in shWTAP and shNC GSCs. (**Q**) WTAP, ROR1, CD31, TJP1 mRNA expression in shWTAP and shNC GSC-2. (**R**) HuR and ROR1 mRNA (left) and protein (right) expression in scramble si, HuR si1, HuR si2 GSCs. Bar chart and line chart data are presented as the mean ± SD or mean ± SEM and were analyzed with Student’s t test or one-way ANOVA. **P* < 0.05, ***P* < 0.01, ****P* < 0.001, and *****P* < 0.0001 for all figures.


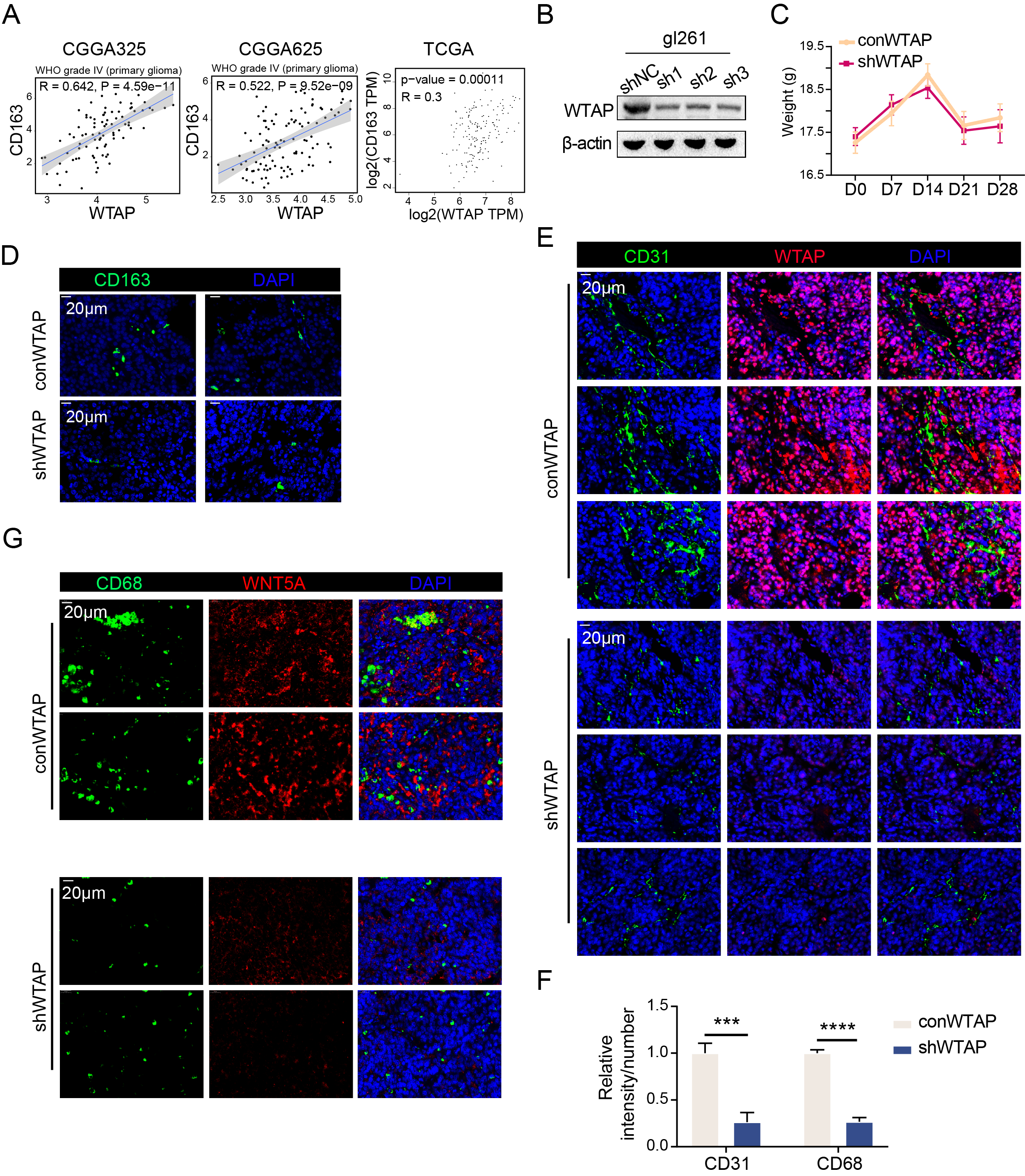


Fig. S10.

**WTAP regulates GDEC differentiation and infiltration of M2 TAMs in vivo.** (**A**) Correlation between expression of WTAP and CD163 in glioma from CGGA and TCGA database. (**B**) WTAP protein expression in shNC, shWTAP_1, shWTAP_2, and shWTAP_3 gl261. (**C**) Weight of tumor-bearing mice from 2 groups. Immunofluorescence staining of CD163 and DAPI (**D**), CD31, WTAP, and DAPI (**E**) in tumor tissues from two groups. (**F**) Relative CD31 expression density and macrophage number quantified by CD68 in tumor tissues between control group and shWTAP group (n=3) (**G**) Immunofluorescence staining of CD68, WNT5A, and DAPI in tumor tissues from two groups. Bar chart data are presented as the mean ± SD and were analyzed with Student’s t test. **P* < 0.05, ***P* < 0.01, ****P* < 0.001, and *****P* < 0.0001 for all figures.


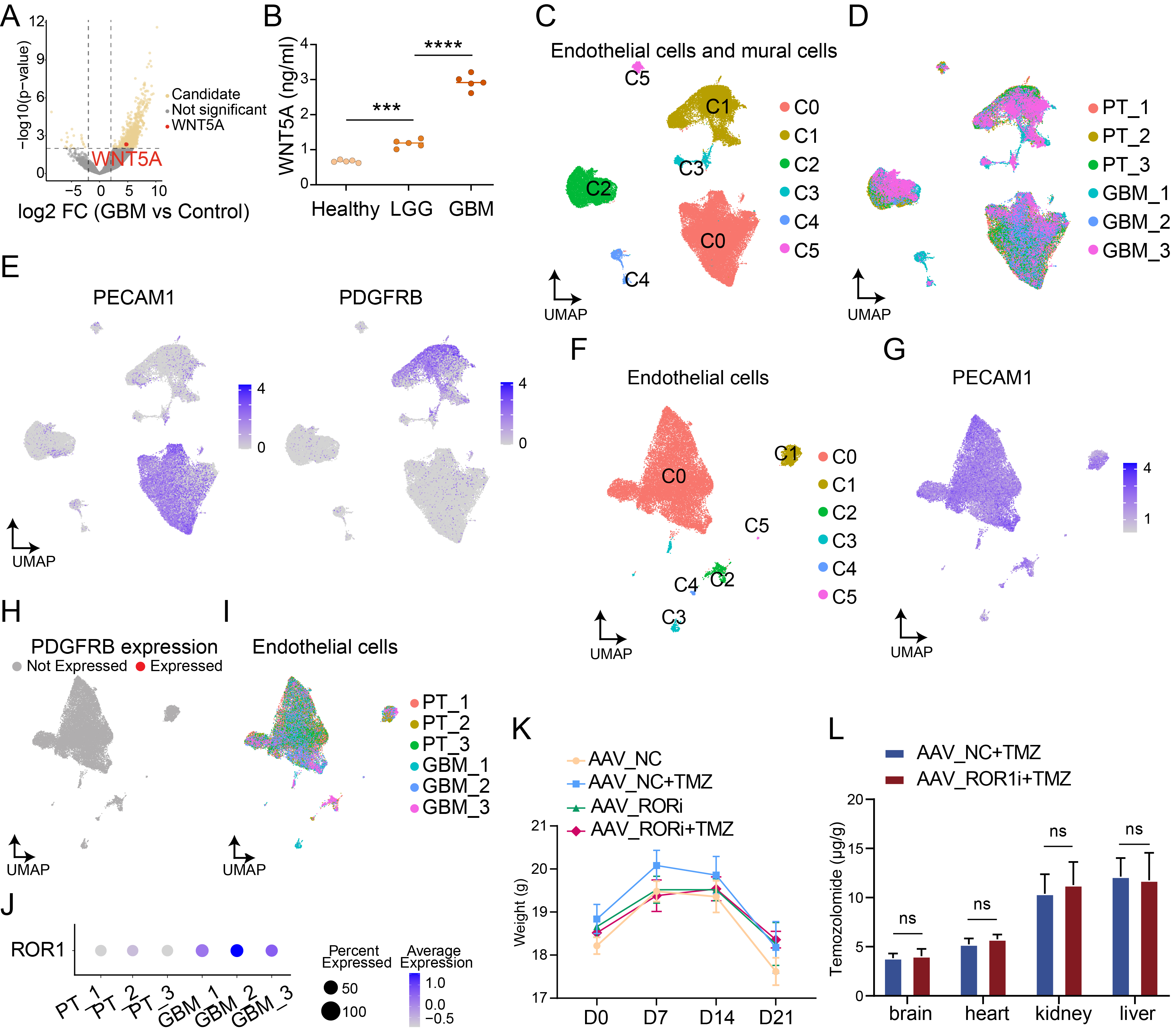


Fig. S11.

**Clinical prospects of ROR1-WNT5A axis in GBM diagnosis and targeted therapy.** (**A**) Differential gene expression analysis of blood samples from six GBM patients versus six healthy controls (GSE106804). (**B**) ELISA analysis of WNT5A levels in peripheral blood samples from GBM patients, LGG patients, and healthy volunteers. (**C**) UMAP plot of endothelial and mural cells divided into 6 clusters from scRNA-seq of 3 GBM tissues and 3 paired peritumoral tissues (GSE242044) by magnetic-activated cell sorting (MACS) depletion of CD45+ leukocytes and enrichment of CD31+ endothelial cells as well as PDGFRβ+ mural cells. (**D**) UMAP plot of endothelial and mural cells annotated by sample origin. (**E**) Feature plot of PECAM1 and PDGFRβ+. (**F**) UMAP plot of endothelial cells divided into 6 clusters after screening by positive expression of PECAM1 and negative expression of PDGFRβ. (**G**) Feature plot of PECAM1. (**H**) Feature plot of PDGFRβ+. (**I**) UMAP plot of scRNA-seq analysis of endothelial cells from GBM tissues and paired peritumoral tissues in 3 patients. (**J**) Dot plot showing expression level of ROR1 in endothelial cells across 3 GBM samples and 3 paired peritumoral samples. (**K**) Weight of tumor-bearing mice from 4 groups. (**L**) Quantification of TMZ concentration in brain, heart, kidney, and liver from tumor-bearing mice in two groups with TMZ treatment. Bar chart data are presented as the mean ± SD and were analyzed with Student’s t test.


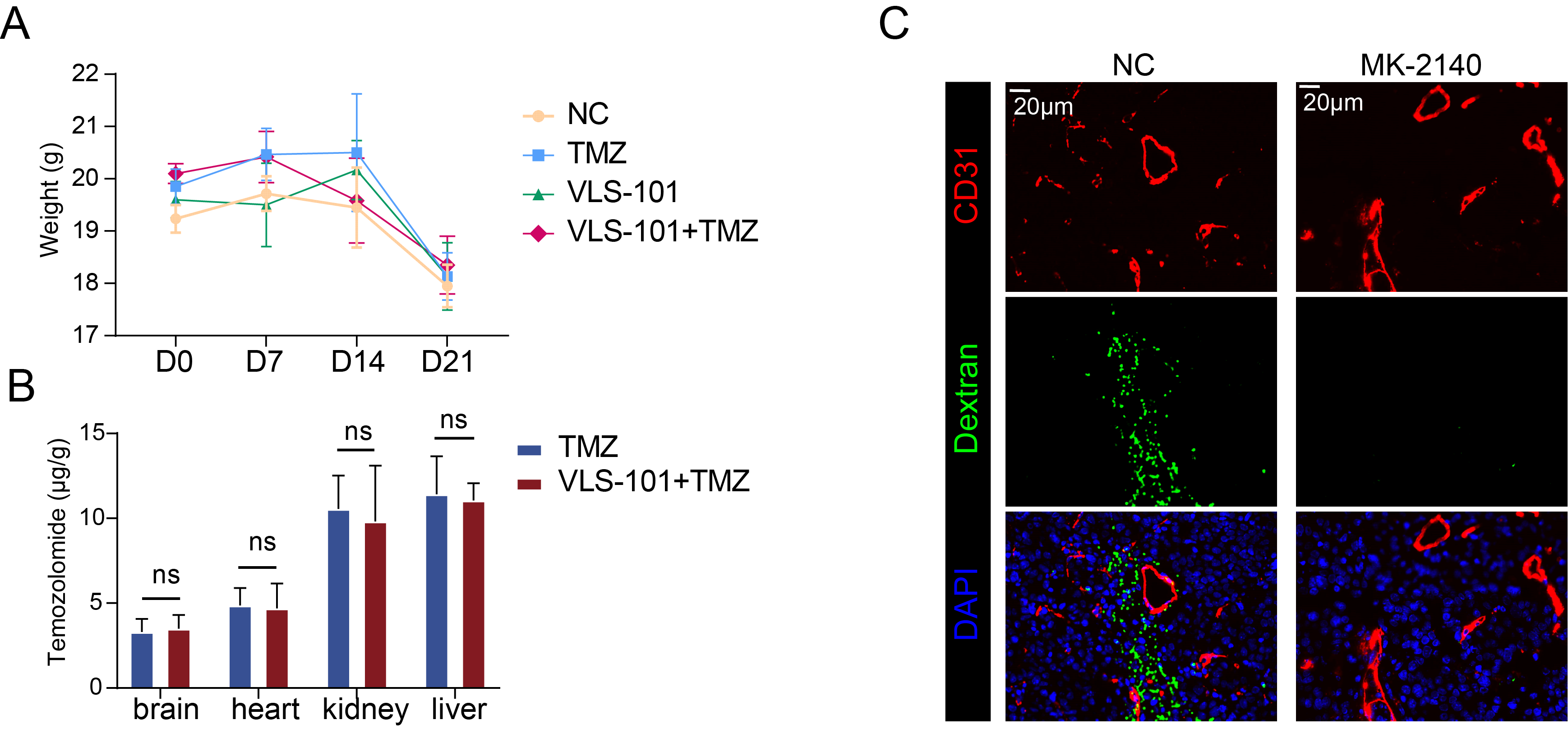


Fig. S12.

**The antibody drug conjugate VLS-101 targeting ROR1 is effective in GBM therapy.** (**A**) Weight of tumor-bearing mice from 4 groups. (**B**) Quantification of TMZ concentration in brain, heart, kidney, and liver from tumor-bearing mice in two groups with TMZ treatment. (**C**) Immunofluorescence staining of CD31, Dextran, and DAPI in GBM tissue from tumor-bearing mice in two groups without TMZ treatment. Bar chart data are presented as the mean ± SD and were analyzed with Student’s t test.

Table S1.

Antibody information

| **Antibody** | **Vendor** | **Catalog Number / RRID** |
| --- | --- | --- |
| CD31 | Proteintech | Cat# 28083-1-AP, RRID:AB_2881055 |
| CD133 | Proteintech | Cat# 66666-1-Ig,RRID:AB_2801586 |
| ROR1 | Cell Signaling Technology | Cat# 16540, RRID:AB_2798764 |
| ROR1 | Proteintech | Cat# 66923-1-Ig, RRID:AB_2882250 |
| β-actin | Proteintech | Cat# 66009-1-Ig, RRID:AB_2687938 |
| TJP1 | Proteintech | Cat# 21773-1-AP, RRID:AB_10733242 |
| WNT5A | abcam | Cat# ab235966, RRID:AB_2894907 |
| WNT5A | Santa Cruz | Cat# sc-365370,RRID:AB_10846090 |
| WTAP | abcam | Cat# ab195380, RRID:AB_2868572 |
| β-catenin | Proteintech | Cat# 51067-2-AP, RRID:AB_2086128 |
| DVL2 | Proteintech | Cat# 12037-1-AP, RRID:AB_2093330 |
| HIF1-α | Cell Signaling Technology | Cat# 36169, RRID:AB_2799095 |
| CD80 | Proteintech | Cat# 14292-1-AP, RRID:AB_10640809 |
| CD206 | Proteintech | Cat# 18704-1-AP, RRID:AB_10597232 |
| CD68 | abcam | Cat# ab303565,RRID:AB_3075482 |
| CD163 | abcam | Cat# ab182422,RRID:AB_2753196 |
| GLUT-1 | abcam | Cat# ab115730,RRID:AB_10903230 |
| HuR | abcam | Cat# ab200342, RRID:AB_2784506 |
| Cleaved Caspase-3 | Cell Signaling Technology | Cat# 9661,RRID:AB_2341188 |

Table S2.

Primer information

| Number | Primer name | Primer sequence (5'to 3') |
| --- | --- | --- |
| 1 | ROR1 (homo) | forward CAGTCAGTGCTGAATTAGTGCC |
|  |  | reverse TCATCGAGGGTCAGGTAAGAAT |
| 2 | 18s rRNA(homo) | forward CGGCGACGACCCATTCGAAC |
|  |  | reverse GAATCGAACCCTGATTCCCCGTC |
| 3 | PECAM1 (homo) | forward AACAGTGTTGACATGAAGAGCC |
|  |  | reverse TGTAAAACAGCACGTCATCCTT |
| 4 | TJP1 (homo) | forward CAACATACAGTGACGCTTCACA |
|  |  | reverse CACTATTGACGTTTCCCCACTC |
| 5 | WNT5A (homo) | forward ATTCTTGGTGGTCGCTAGGTA |
|  |  | reverse CGCCTTCTCCGATGTACTGC |
| 6 | CD80 (homo) | forward GGAATACAACCAAGCAAGAGCAT |
|  |  | reverse TAGGTCAGGCAGCATATCACAA |
| 7 | CD206 (homo) | forward TGGTGAACGGAATGATTGTGTAG |
|  |  | reverse GGTCCATCTTCCTTGTGTCAG |
| 8 | WTAP (homo) | forward CTTCCCAAGAAGGTTCGATTGA |
|  |  | reverse TCAGACTCTCTTAGGCCAGTTAC |
| 9 | HIF1A (homo) | forward GAACGTCGAAAAGAAAAGTCTCG |
|  |  | reverse CCTTATCAAGATGCGAACTCACA |

Table S3.

Targets sequence

| Targets | Senquenes (sense strand) |
| --- | --- |
| HuR siRNA 1 (human) | CGAAGCCUGUUCAGCAGCATT |
| HuR siRNA 2(human) | UAAAGUAGCAGGACACAGCTT |
| Scramble siRNA(human) | UUCUCCGAACGUGUCACGUTT |
| ROR1 shRNA1(human) | CCGTACTGCGATGAAACTTCA |
| ROR1 shRNA2(human) | GCAAGATCAAATCCCATGATT |
| ROR1 shRNA3(human) | GGAACATCTCAAGTGAACTCA |
| WTAP shRNA1(human) | ATGGCAAGAGATGAGTTAATT |
| WTAP shRNA2(human) | GTTATGGCAAGAGATGAGTTA |
| WTAP shRNA3(human) | GCAAGAGTGTACTACTCAAAT |
| NC shRNA(human) | CCTAAGGTTAAGTCGCCCTCG |
| WTAP shRNA1(mouse) | CCTGGAAGTTTACGCCTGATA |
| WTAP shRNA2(mouse) | GCACGGGATGAGTTAATTCTA |
| WTAP shRNA3(mouse) | GACCCAGCAATCAACTTGTTT |
| NC shRNA(mouse) | CCTAAGGTTAAGTCGCCCTCG |
| AAV_ROR1i(human) | GGAGAATGTCCTGTGTCAAAC |
| AAV_NC(human) | CGCTGAGTACTTCGAAATGTC |
